# Enhanced EPR directed and Imaging guided Photothermal Therapy using Vitamin E Modified Toco-Photoxil

**DOI:** 10.1038/s41598-018-34898-3

**Published:** 2018-11-12

**Authors:** Deepak S. Chauhan, Amirali B. Bukhari, Gayathri Ravichandran, Ramkrishn Gupta, Liya George, Radhika Poojari, Aravind Ingle, Aravind K. Rengan, Asifkhan Shanavas, Rohit Srivastava, Abhijit De

**Affiliations:** 1 Department of Biosciences and Bioengineering, Indian Institute of Technology Bombay, Powai, Mumbai, India; 2 Molecular Functional Imaging Lab, Advanced Centre for Treatment, Research and Education in Cancer (ACTREC), Tata Memorial Centre, Kharghar, Navi Mumbai, India; 3 Laboratory Animal Facility, Advanced Centre for Treatment, Research and Education in Cancer (ACTREC), Tata Memorial Centre, Kharghar, Navi Mumbai, India; 4 Department of Biomedical Engineering, Indian Institute of Technology Hyderabad, Hyderabad, India; 5 Institute of Nano Science and Technology, Mohali, Punjab India

## Abstract

Herein we report synthesis, characterization and preclinical applications of a novel hybrid nanomaterial Toco-Photoxil developed using vitamin E modified gold coated poly (lactic-co-glycolic acid) nanoshells incorporating Pgp inhibitor d-α-tocopheryl polyethylene glycol 1000 succinate (TPGS) as a highly inert and disintegrable photothermal therapy (PTT) agent. Toco-Photoxil is highly biocompatible, physiologically stable PTT material with an average diameter of 130 nm that shows good passive accumulation (2.3% ID) in solid tumors when delivered systemically. In comparison to its surface modified counterparts such as IR780-Toco-Photoxil, FA-Toco-Photoxil or FA-IR780-Toco-Photoxil accumulation are merely ~0.3% ID, ~0.025% ID and ~0.005% ID in folate receptor (FR) negative and positive tumor model. Further, Toco-Photoxil variants are prepared by tuning the material absorbance either at 750 nm (narrow) or 915 nm (broad) to study optimal therapeutic efficacy in terms of peak broadness and nanomaterial’s concentration. Our findings suggest that Toco-Photoxil tuned at 750 nm absorbance is more efficient (P = 0.0097) in preclinical setting. Toco-Photoxil shows complete passiveness in critical biocompatibility test and reasonable body clearance. High tumor specific accumulation from systemic circulation, strong photothermal conversion and a very safe material property in body physiology makes Toco-Photoxil a superior and powerful PTT agent, which may pave its way for fast track clinical trial in future.

## Introduction

Nanomaterials are being utilized in diverse field like electronics, magnetics, optoelectronics, biomedicines, cosmetics and other areas. For cancer cure, nanomaterials provide new dimensions for intervening tumor growth by precisely controlling and delivering the therapeutic dosage to the required target^1^. Over the past few years, photothermal therapy (PTT) has emerged as a promising alternative for spatially controlled treatment option for localized cancer. The knowledge of non-harmful nature of near infra-red (NIR) light in tissue environment when combined with plasmonic nanomaterial provide localized heat based on plasmon resonance principle^2^. Use of such plasmonic heat has been the basis for localized and safe treatment option in locally advanced tumors. Various photothermal agents have been fabricated like the carbon nanotubes^3^, gold nanorods^4^, nanoshells^5,6^, *etc*. Gold nanoshells have shown promises as effective PTT agents because of their ability to tune the absorbance peak at NIR wavelength by varying the core to shell ratio and their increased biocompatibility in comparison to other photothermal agents^7^. Oldenberg *et al*. and others have exploited the properties of the gold shell formed over silica core and other dielectric core by tuning the absorbance to the infrared region^8–10^. Previously, we have also reported design and synthesis of biodegradable, thermos-labile liposome gold hybrid nanostructures (LiposAu) for PTT^11,12^. However, the non-biodegradable nature of silica-gold hybrid nanoshells poses great concern in terms of their elimination from the body while the low stability of LiposAu at physiological condition makes intra-tumoral delivery of the material as the only choice. Due to these limitations, their potential as PTT agent in the cancer clinic is limited. Looking at the demands of an ideal PTT agent for human use, a material must present a balanced combination of characters such as biocompatibility, biodegradability, good body clearance, effective accumulation at the target tumor site and most importantly a high photothermal conversion capacity for a standalone PTT. Hence, the need for a disintegrable as well as more physiologically stable material like poly (lactic-co-glycolic acid) (PLGA, a FDA approved polymer) and chitosan that can be hybridized with gold remains justified. In terms of biocompatibility and biodegradability, similar to the lipid or silica, PLGA and chitosan have been extensively delved in the field of nano-drug delivery^13^.

Despite the tremendous growth in fabrication of nanomaterials for healthcare and cancer theranostic, there is lack of information over the toxicity hazards that usually lead to failure in clinical trial. If the commercialization of nanomaterials for healthcare, especially parenteral administration is anticipated, a thorough biocompatibility and systemic toxicity study is critically required^14^. Enlightened by previous literature where multifunctional gold-coated and also drug loaded PLGA nanoshells have been synthesized for compensating the therapeutic effect of photothermal therapy with chemotherapeutic drugs^8,9^. It’s has been evidently noticed that complexity in nanomedicine design has clearly complicated their path towards successful clinical application. For example, actively targeted BR96-doxorubicin showed good efficacy in mouse tumor models but caused significant toxicity in phase II clinical trial. It could be explained as specificity in animals’ models may not necessarily predict the specificity in human trials^15^. Thus, it could be advantageous to avoid unnecessary complexities in material fabrication and rigorously test the toxicity of nanomaterials in physiological setting. It should also be noted that intravenously injected nanomaterials encounters multiple line of body defense to neutralize and eliminate the substance. Adsorption of plasma protein over the surface is the first barrier and cause aggregation of nanomaterials and activation of various defense cascades to eliminate the particles from blood stream that ultimately reduces the % accumulation in the targeted area. Review of fundamental physiochemical properties (size, charge, stability, and surface modification) is critical as these factors immensely affect the nanomaterials biodistribution, transport across vascular walls as well as retention in tumor.

Keeping these viewpoints, in this study we have fabricated a disintegrable and highly inert TPGS incorporated PLGA nanoshells and tested its effectiveness as a stand-alone photothermal therapy agent in preclinical model. Utmost care has been taken in thorough material characterization and biocompatibility evaluations to address all probable safety concerns of clinical significance. The relevance of narrow vs. broad NIR peak absorbance as well as passive vs. active targeting of the nanomaterial for obtaining highest therapeutic efficacy within a low laser power setting is presented here. Body clearance issue of gold nanoparticles is addressed by using non-invasive NIR fluorescence (NIRF) imaging, inductively coupled plasma mass spectrometry (ICP-MS) and transmission electron microscope (TEM) analysis of tissue samples. Further, inclusion of critical toxicity studies like hemolysis, reactive oxygen species (ROS) measurement, apoptosis alongside full spectrum biochemical assessments are some of the critical aspects presented here.

## Methods

All animal procedures were carried out in accordance with the experimental protocols approved by the Institutional Animal Ethics Committee, ACTREC, India. A minimum of 3 mice were used per treatment groups, and were randomly assigned similar to our previous study^12^. No outliers were excluded and histology images were analyzed by pathologist blinded to study.

### Synthesis of Toco-Photoxil

Toco-Photoxil was synthesized in two steps. First, glycol chitosan functionalized PLGA NPs were prepared using the solvent evaporation method. Second, gold seeds were reduced over the surface of glycol chitosan functionalized PLGA NPs using *ex-situ* method. Detailed methodology is provided in Supplementary Information.

Folic acid was conjugated to Toco-Photoxil via the glutathione. Folic acid-glutathione conjugate was prepared using the EDC/NHS crosslinking. The conjugate was attached on the surface of Toco-Photoxil via thiol group. Detailed methodology is provided in Supplementary Information.

### Disintegration of Toco-Photoxil

To determine the disintegration ability of Toco-Photoxil after photothermal treatment, 100 µl of 50 µg/ml Toco-Photoxil (750 nm) were kept at 37 °C in a water bath and then irradiated with 750 nm laser for varying time periods. Samples were analyzed by UV-Vis spectroscopy and FEG-TEM.

### 2D and 4D X-ray imaging

To determine the potential of Toco-Photoxil as a contrast agent- X-ray and four-dimensional X-ray imaging (FDXM) studies were conducted. Toco-Photoxil (1 mg), iodine (5 mg) and negative control (Milli-Q) were taken in Eppendorf tubes and exposed to X-ray using Siemens X-ray Digital Machine. While four-dimensional X-ray imaging was performed via 1% agarose phantoms using ZEISS Xradia 520. FDXM images were further processed with the help of ImageJ and Fiji.

### *In vitro* assessment of Toco-Photoxil biocompatibility

The biocompatibility of Toco-Photoxil was assessed on L929 and NIH3T3 (procured from NCCS Pune, India) cell lines. 200 µl of different (25 µg/ml to 125 µg/ml) concentration of Toco-Photoxil was added to the cells seeded in 96 well plates. After 24 h, supernatant was discarded and MTT assay was performed. Detailed methodology is provided in Supplementary Information.

The ROS index of Toco-Photoxil and FA-Toco-Photoxil was determined using the (5-(and-6)-chloromethyl-2′,7′-dichlorodihydrofluorescein diacetate, acetyl ester) (CM-H2DCFDA) dye. Detailed methodology is provided in Supplementary Information.

### Hemolysis study

150 µl of RBCs was added to 750 µl (125 µg/ml and 50 µg/ml) of Toco-Photoxil and the mixture was incubated for 1 h and 24 h at 37 °C. After incubation, the mixture was pelleted down at 15000 rpm, and the absorbance of supernatant containing hemoglobin was recorded using TECAN Pro plate. 750 µl of water and PBS were used as positive and negative control. For SEM imaging, all procedure was same as above except samples were pelleted down at 2000 rpm and RBCs were fixed with 2.5% of glutaraldehyde. Detailed methodology is provided in Supplementary Information.

### Biodistribution and histopathological evaluation

Nude mice of 6–8 weeks weighing around 20 g were placed in two different Groups. Control-Animals were injected with normal saline, and Test-Animals were injected with 200 µl of 125 µg/ml Toco-Photoxil and FA-Toco-Photoxil dispersed in saline solution via the tail vein. The dose chosen was determined by *in vitro* MTT based toxicities assay performed over L929 cells.

Each group of animals was sacrificed at predetermined time intervals by cervical dislocation; blood samples were collected by cardiocentesis and major organs like liver, spleen, kidney, lung, and muscles were harvested, observed grossly and weighted. To determine the amount of accumulation of Toco-Photoxil in various major organs, ICP-MS was used. For ICP-MS, samples were prepared as per our earlier report^12^. Coefficient of organs was also determined by dividing the weight of organs (mg) to whole body weight (gms). For Histopathology evaluation, part of major organs harvested was immediately stripped and fixed in 10% formalin, later embedded in paraffin block and 5 μ thin sections were cut for staining using Hematoxylin and Eosin (H&E) before analyzing using optical microscope.

### Hematology and serum biochemical analysis

Blood was collected from the control and test group animals on the 28^th^ day group in EDTA coated vial for hematology, and in normal sterile vial for serum biochemical analysis. Blood was allowed to clot at room temperature and the serum obtained above was given for analysis to Unique Bio-diagnostic Enterprises (UBE), Mumbai, India. MYTHIC 18 Cell counter was used for hematology and EM 200 Biochemistry analyzer-TransAsia for serum biochemical analysis.

### Photothermal transduction experiment

The photothermal effect of the prepared nanostructures was studied using 750 nm and 915 nm NIR laser of power 650 mW/cm^2^. 100 µl of 25–125 µg/ml of Toco-Photoxil tuned at 750 nm, 915 nm and control solutions (Milli-Q and GCHT-PLGA) were added in a 96 well plate and allowed to float on a water bath at 37 °C. Controls and test samples were kept widely separated to avoid the heat transfer. The temperature increment was recorded using an infrared digital thermometer (Oakton Mini-InfraPro, Cole-Parmer, India). Additional information is provided in Supplementary Information.

### *In vitro* assessment of Toco-Photoxil directed photothermal therapy

For *in vitro* photothermal treatment, 5 × 10^4^ MCF-7, MDA-MB-231, SKBR3, HeLa and HT1080 cells were seeded in a 96-well plate. 100 µl of 50 µg/ml of 750 nm tuned Toco-Photoxil were added and the respective wells were exposed to 750 nm NIR light for 4 min. and then again incubated at 37 °C with 5% CO_2_ for 24 h. Next day, the wells were processed for the formation of blue formazan crystals and the calorimetric measurements were carried out as mentioned above. All measurements were done in triplicates. For qualitative assessment of photothermal therapy and for the determination of the distribution of cell cycle after photothermal treatment, same procedure was followed as explained above except after 24 h, propidium iodide (PI) solution (5 µg/ml) was added and left for 15 min. before analyzing using FACSVerse Flow Cytometer and Nikon Eclipse Ti fluorescence microscope at Ex = 488 and Em = 580 nm. Data were analyzed using FlowJo 10. MCF-7 cells unstained untreated and stained untreated were used as controls.

### *In vivo* bioluminescence imaging and photothermal therapy

Cells inoculation and bioluminescence imaging were performed as previously described^12^. Briefly, 1 × 10^6^ HT1080-fluc2-turboFP cells were injected subcutaneously into the right flank of female BALB/c NUDE mice. Assessment of cells injection was monitored using bioluminescence imaging after intraperitoneal injection of 3 mg/100 µl/mouse of D-luciferin substrate. Animals were anesthetized using 1–2% isoflurane and imaged using IVIS Spectrum (Perkin Elmer, USA) imaging system at an exposure setup of 10 seconds, medium binning, and f-stop (f) = 1.

At day 20, mice were randomly segregated into control (n = 4; group I), 750 nm laser control (n = 4; group II), 915 nm laser control (n = 4; group III), 750 nm laser + Toco-Photoxil tuned at 750 nm (n = 4; group IV), and 915 nm laser + Toco-Photoxil tuned at 915 nm (n = 4; group V). On day 24, mice were either administered intratumorally with 30 µl of normal saline or ~20 µg/30 µl of 750 nm tuned Toco-Photoxil or 915 nm tuned Toco-Photoxil. Tumors were then treated by irradiating either 750 nm or a 915 nm fixed wavelength laser for 4 min. on laser alone groups or Toco-Photoxil injected photothermal treatment groups. Pre- and post-therapy, bioluminescence imaging was performed to monitor the efficacy of the photothermal therapy. Luminescence light output was quantified in terms of average radiance (p/sec/cm^2^/sr) using the Living Image v4.4 software. The pseudocolor bar represents the photons captured by the scanner.

### *In vivo* near infrared fluorescence (NIRF) imaging and photothermal therapy

For surface functionalization and NIRF imaging, Toco-Photoxil and FA-Toco-Photoxil were tagged with hydrophobic IR780 dye. Two types of tumor models used for this study i.e. folate receptor positive orthotopic (4T1) and folate receptor negative/low xenograft (HT1080). The density of folate receptors on these cell lines was also compared with the help of ICP-MS. Briefly, equal number of cells were cultured in 12 well plate. Thereafter, equal amount of FA-Toco-Photoxil was added and after 24 h, supernatant was discarded and cells were washed thrice to remove all unbound particles. Cells were trypsinzed and the gold content was analyzed with the help of ICP-MS.

Once the tumor reached to size of 100 mm^3^, mice were segregated into the dye alone group (n = 2) and the dye-tagged Toco-Photoxil and FA-Toco-Photoxil groups (n = 3 each). All mice were intravenously injected with respective materials as above. Animals were anesthetized after 1 h, and NIRF imaging was performed using the IVIS Spectrum imaging system using an excitation bandpass filter of 745 nm and an emission bandpass filter of 800 nm. The images were acquired at an exposure time of 1 second, medium binning, and f-stop 4. Images were acquired at 1, 4, 12, 24, 48, and 72 h post injection. Photothermal therapy was performed for two consecutive days (between 24–72 h post-injection time points). TurboFP (580 nm excitation and 640 nm emission bandpass filters) fluorescence imaging was also performed for post-therapy follow-up at various time points. Fluorescence light output was quantified in terms of average radiant efficiency [(p/sec/cm^2^/sr)/(µW/cm^2^)] using the Living Image v4.4 software.

### Statistical analysis

All viability, histology, photothermal efficiency data were normalized to controls. The data are presented as means with standard deviation. Statistical analysis along with graphs generation was done in GraphPad Prism 6.0 software for Windows. The ANOVA with Tukey’s multiple comparison test and Student’s t test was performed. All significant pairwise comparison is shown and specific p-value indicated in the figure caption. No outliers were excluded and data was considered significant at *P < 0.05, **P < 0.01, and ***P < 0.001.

## Results

### High yield synthesis of vitamin E modified PLGA nanoparticles

**Figure 1:**
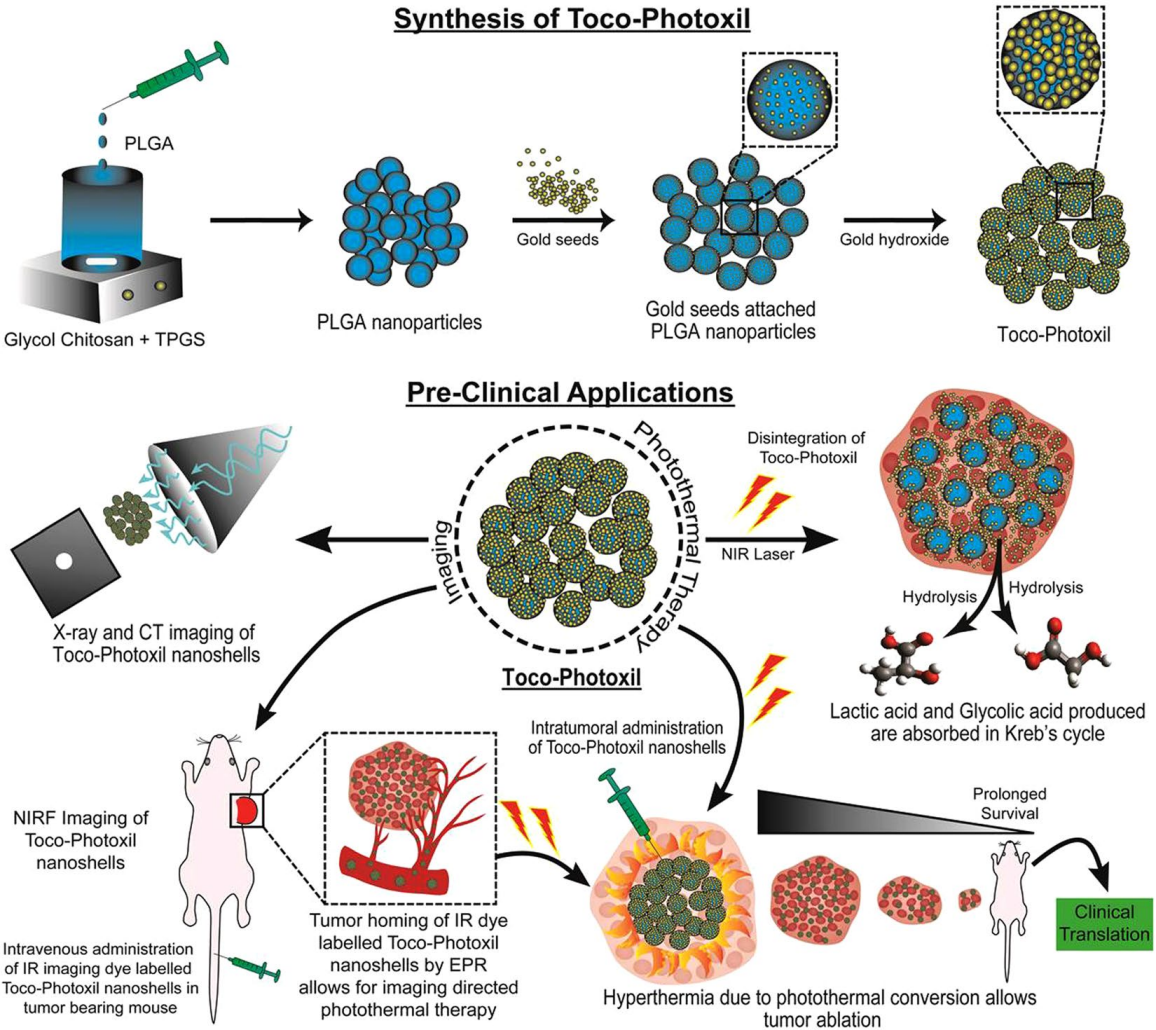
Illustration showing the synthesis and preclinical applications of Toco-Photoxil for imaging guided photothermal therapy.

**Figure 2:**
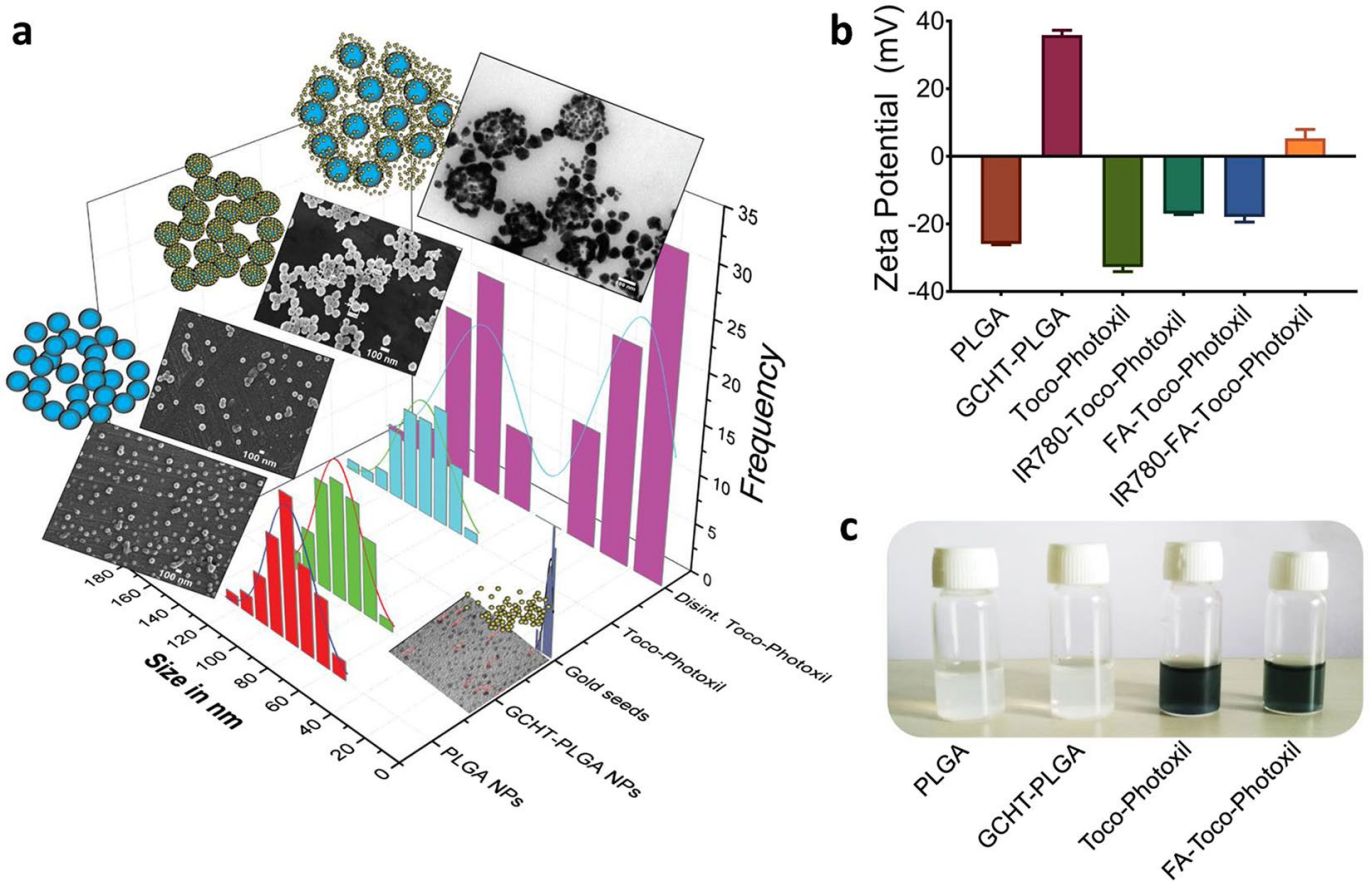
Characterization of nanoformulations. (**a**) Size distribution of nanomaterials synthesized at different stages overlapped with their representative FEG-SEM and FEG-TEM images. (**b**) Graph shows the zeta potential values of the various nanoformulations. (**c**) Representative photograph of PLGA NPs, GCHT-PLGA NPs, Toco-Photoxil, and FA-Toco-Photoxil nanoformulations.

PLGA nanoparticle was prepared using acid terminated PLGA (molecular weight − 17000 Da) and 50:50 lactide to glycolide ratio. This configuration was chosen due to its potential for rapid breaking down into lactic and glycolic acid which are metabolized in the body^13,16^. PLGA nanoparticles were prepared by an emulsion-solvent evaporation method using antibacterial and antifungal agent d-α-tocopheryl polyethylene glycol 1000 succinate (TPGS, an FDA-approved vitamin E modified emulsifier) (Fig. 1). Additionally, the use of TPGS drastically increased the yield of nanoshells to almost 100% when compared to our earlier attempt using emulsifier poly (vinyl alcohol) (PVA) giving a <10% yield. Hydrodynamic diameter determined by dynamic light scattering (DLS) was ~142.2 nm and size determined using scanning electron microscope (SEM) found to be ~100 nm (Fig. 2a and Supplementary Fig. S1a).

### Surface functionalization with cationic polymer

**Figure 3:**
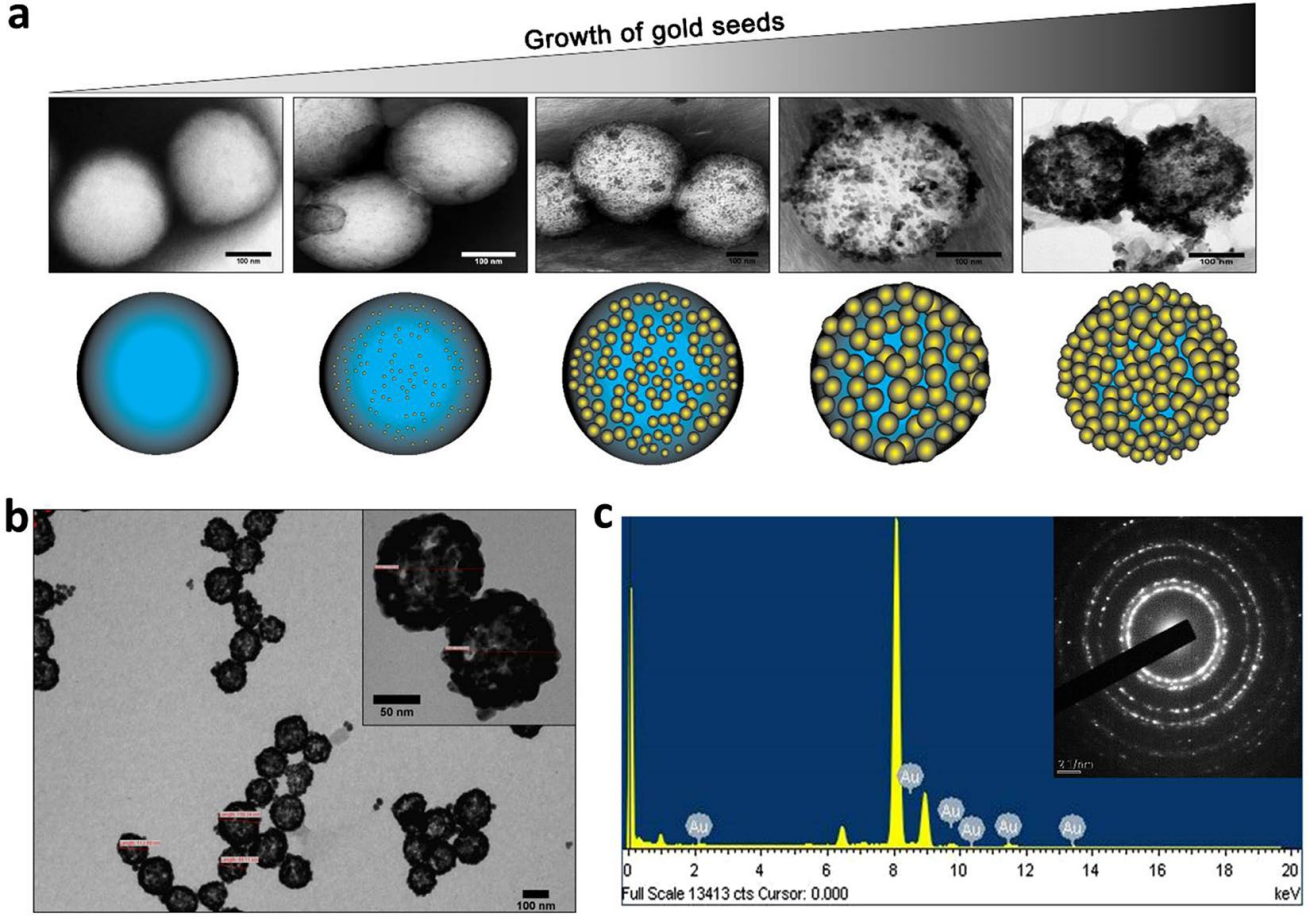
Gold coating *via* seed-growth mediated method. (**a**) FEG-TEM images of Toco-Photoxil captured at different time period during the synthesis process. (**b**) FEG-TEM image of Toco-Photoxil synthesized using glycol chitosan. (**c**) EDAX and diffraction pattern (inset) of Toco-Photoxil showing the presence of gold shell.

PLGA nanoparticle (zeta potential −25.03 ± 1.10 mV) surface was functionalized with amine groups using glycol chitosan (GCHT) for the attachment of negatively charged gold seeds *via* electrostatic force^9^. GCHT coating on the particle was confirmed as the zeta potential changed from −25.03 ± 1.10 mV to +36.35 ± 1.55 mV (Fig. 2b). Further, stretching of the amide bond of GCHT at 1626 cm^−1^ was present and also the intensity of C = O stretching due to PLGA ester at 1756 cm^−1^ was found to be less intense than pure PLGA (Supplementary Fig. S3a). Both chitosan and glycol chitosan were used for rendering positive charge over PLGA nanoparticles. The usage of chitosan resulted in the formation of about 200 nm sized nanoparticles, which we suspect due to the requirement of acidic pH for the formulation (Supplementary Fig. S1c). While in case of GCHT, the size obtained is about 120 nm (Figs 2a, 3b and Supplementary Fig. S1b). GCHT was also preferred over the other cationic polymers because of its water soluble nature at neutral pH. In addition to this, the ability of GCHT to generate small sized PLGA nanoparticles also potentiate enhanced X-ray attenuation for imaging, and enhanced permeability and retention (EPR) effect^17,18^.

### Highly stable as well disintegrable gold shell formation and tuning in NIR region

PLGA nanoparticles combined with GCHT exposed the positively charged amine groups, thus facilitating attachment of negatively charged gold seeds. Gold seeds were prepared by the reduction of chloroauric acid with tetrakis (hydroxymethyl) phosphonium chloride (THPC) which resulted in the formation of ~3 nm size seeds having zeta potential −12 ± 1.84 mV (Fig. 2a and Supplementary Fig. S1d). Seeds were then attached to the amine group of GCHT *via* co-ordinate bonding and enhanced through electrostatic force at pH 6.5. Gold seeds acted as nucleation center in the formation of a thin layer of gold nanoparticles over the PLGA core with the help of gold hydroxide (Fig. 3a).

Toco-Photoxil formed using THPC was found to be more stable as compared to the citrate method of gold coating^19^ and its zeta potential was determined to be −31.82 ± 1.62 mV (Fig. 2b). Gold coating was confirmed using transmission electron microscope (TEM) (Fig. 3b), as well as energy dispersive X-ray spectroscopy (EDAX) and selected area electron diffraction (SAED) analysis (Fig. 3c).

Further, to determine the presence as well as nature of gold coating, X-ray photoelectron spectroscopy (XPS) was performed. In comparative spectral analysis, the peaks of gold (centers around 83–90 eV) were explicitly observed in Toco-Photoxil with respect to the positive (e.g. chloroauric acid) and negative (e.g. PLGA NPs) control (Supplementary Fig. S2a). The in between steps of gold reduction during the shell formation was also investigated. It was observed that peaks of gold shifted to lower energy as the reduction steps preceded. In chloroauric acid, the peaks of gold found to be centered around 90, 86 and 83 eV while in Toco-Photoxil peaks shifted to lower energy i.e. 87 and 83 eV. The gold seeds attached with PLGA NPs (intermediary sample) show the shift in peaks in between of chloroauric acid and Toco-Photoxil, indicating *ex-situ* (step-wise) nature of gold coating (Supplementary Fig. S2b). The surface of Toco-Photoxil was further analyzed for the presence and percentage composition of other elements. In the survey spectrum, the percentage composition of Au, C, O and N were found to be in the ratio of 70.96, 12.81, 15.60, and 0.63 respectively, which is also concordant with the EDAX analysis (Supplementary Fig. S2c–f).

The hydrodynamic diameter of Toco-Photoxil was determined to be ~155 nm and size determined by SEM and TEM was ~130 nm (Figs 2a, 3b, and Supplementary Fig. S1e). The meticulous removal of excess gold seeds was critical, which otherwise grow into gold nanoparticles. The gold layer formation over the PLGA core-shell shifts the gold plasmon resonance from visible to NIR range. The peak absorbance was tuned to the desired NIR wavelengths by varying the amount of gold hydroxide; as in the case of nanoshells, the peak absorbance shift to NIR relates to the ratio of core to shell diameter. Herein, the NIR peak was tuned at two different wavelengths of varying width i.e. 750 nm (narrow) and 915 nm (broad) to compare therapeutic efficacy.

To determine the disintegration ability of Toco-Photoxil, UV-Vis absorbance was measured after laser irradiation and the structural integrity of shell was determined by field emission gun-transmission electron microscope (FEG-TEM). It was observed that the peak absorbance of Toco-Photoxil gradually shifts towards lower absorbance as the laser irradiation time increases (data not shown). The non-irradiated vs. laser irradiated Toco-Photoxil sample was analyzed by FEG-TEM, wherein disintegration of gold shell was observed suggesting their hydrolysis assisted photo-disintegrable nature (Fig. 2a and Supplementary Fig. S1f).

### One step folic acid conjugation

Folic acid (FA) was conjugated to Toco-Photoxil via antioxidant glutathione (GSH) to specifically target the over-expressing folate receptors of cancer cells. Instead of attaching glutathione and folic acid stepwise, GSH-FA conjugate was prepared as one step attachment process, thus minimizing loss of yield in repeated washing steps. GSH-FA conjugate was prepared as explained in materials and method, after preliminary confirmation of conjugate using UV and FTIR, 1H NMR established covalent bonding between the GSH and FA. In GSH-FA conjugate spectra, typical FA peaks at 6.54 (d, 3, 5-H of FA), 4.48 (m, α-CH2 of Glu of FA, 1H) and peaks attributed to aromatic protons singlet at 8.4 ppm, doublet at 7.5 ppm and 6.7 ppm are presents along with peaks of GSH at 2.03–2.08 (m, 3H); 2.40–2.46 (m, 3H), 2.82 (br d, J = 6.16 Hz, 3H), 3.85 (s, 3H), 4.68 (s, 6H). The appearance of these peaks confirms the successful conjugation of glutathione and folic acid (Supplementary Fig. S3b–d). It was found that 27 µg/ml of folic acid was attached over the surface of Toco-Photoxil and the yield of FA-Toco-Photoxil did not reduce drastically as that was the case in step-wise conjugation attempted previously. (Supplementary Fig. S4a).

The number of FA molecules attached to Toco-Photoxil was also determined, it was found that the effective number of FA molecules per conjugate to be ~ 4.1 × 10^6^. Detailed calculation method followed is provided in Supplementary Section.

### Evidence of low oxidative index and metabolic inertness

To determine the ROS index of Toco-Photoxil, 5-(and-6)-chloromethyl-2′,7′-dichlorodihydrofluorescein diacetate acetyl ester (CM-H2DCFDA) dye was used which is oxidized to green fluorescein- 2′,7′-dichlorofluorescein (DCF) by intracellular oxidants (primarily hydrogen peroxide). It was found that Toco-Photoxil and FA-(GSH)-Toco-Photoxil did not induce any significant ROS in cells incubated for 24 h (Supplementry Fig. S5a). Noteworthy, ROS level induced by Toco-Photoxil and FA-(GSH)-Toco-Photoxil are nearly the same and even less than the unstained, untreated control and markedly lower than the H_2_O_2_ treated positive control.

To further determine the biocompatibility of Toco-Photoxil in terms of metabolic activity of cells, two mouse normal fibroblast cell lines (L929 and NIH3T3) were used. Cells were incubated with varying concentration of particles (25–125 µg/ml) for 24 h. It was observed that cells retained more than 90% viability at the highest concentration (125 µg/ml) tested (Supplementary Fig. S5b).

### Hemolytic potential and acute toxicity response

**Figure 4:**
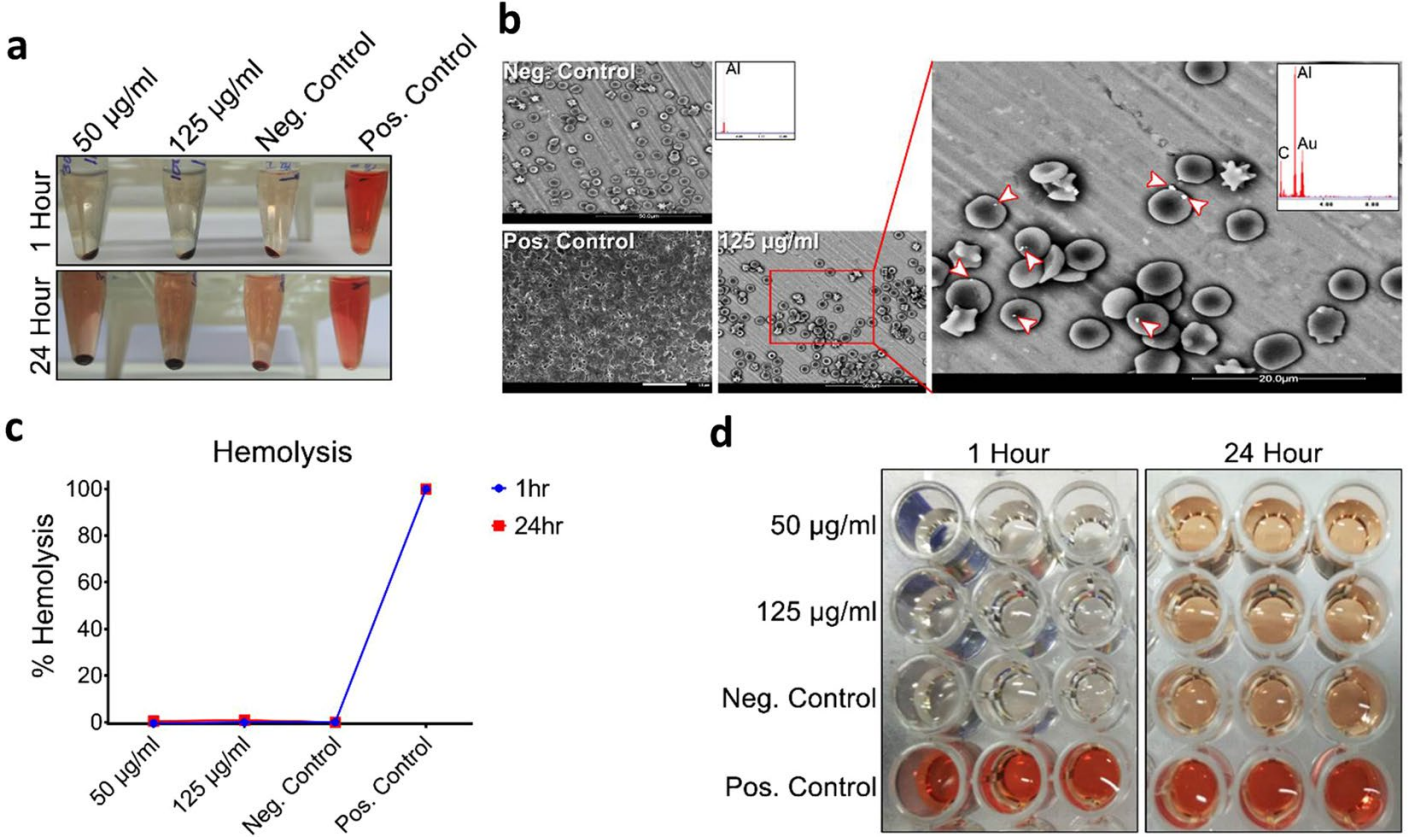
Assessment of Toco-Photoxil on haemolysis. (**a**) Photographic images of RBC suspended with different concentrations (µg/ml) of Toco-Photoxil, PBS (negative control), and water (positive control) at 1 h and 24 h time period. (**b**) ESEM images of RBCs treated with PBS (negative control), water (positive control), and 125 µg/ml of Toco-Photoxil. Arrow heads indicate the presence of Toco-Photoxil over RBCs as confirmed by EDAX analysis. (**c**) Graph shows the percentage hemolysis. (**d**) Photographic images of supernatant used for recording the absorbance at 577 nm.

Hemolysis is another common physiological acute toxicity response that ensures the biocompatibility of intravenous injectable nanomaterials. On this account, a hemolysis assay was set out to assess the physiological compatibility of Toco-Photoxil in mice. As can be seen that two different concentrations, a therapeutic dosage (50 µg/ml) and more than double the therapeutic dosage (125 µg/ml) were tested, but none caused any hemolysis effect within 24 h of incubation (Fig. 4c). The hemolytic potential of Toco-Photoxil was found almost equal to the negative control i.e. red blood cells (RBCs) incubated with PBS. It could also be seen in photographic images taken after pelleting down the RBCs and nanoparticles that there was no hemolysis even after 24 h of Toco-Photoxil incubation in respect to positive control (e.g. water) where distinctive red color is observed as an indicator of almost 100% hemolysis (Fig. 4a,d). Percentage of hemolysis was calculated using the following equation by recording the absorbance of released hemoglobin at 577 nm and subtracting it with absorbance at reference wavelength 655 nm:

$$(Toco{\textstyle \text{-}}Photoxil\,absorbance\,-\,negative\,control\,absorbance)/(positive\,control\,absorbance\,-\,negative\,control\,absorbance)\times 100$$

The probability of false positive results was also controlled by incubating the Toco-Photoxil with the hemoglobin released in positive control and subsequently pelleting down at 15000 rpm and then recording the absorbance at 577 nm with reference at 655 nm. No significant decrease in absorbance before and after incubation with the Toco-Photoxil suggests non-adsorbent and safe nature of Toco-Photoxil towards hemoglobin.

The interaction of Toco-Photoxil with RBCs was also determined using environmental scanning electron microscope (ESEM), and as can be seen in images captured in backscattering mode that there is very less affinity (i.e. very less number of particles found over surface of RBCs membrane) of Toco-Photoxil’s towards RBCs, and after 24 h incubation there was no change in morphology observed in RBC in comparison to negative and positive control (Fig. 4b).

### Toco-Photoxil demonstrates reasonably good body clearance

Although, nanoparticle-mediated treatments are advancing at fast rate, there is always a grave concern whether it accumulates in the body or gets cleared in a matter of time. In this regard, the presence of gold in major organs was measured on 1, 4 and 28 days after intravenous injection using ICP-MS. It was observed that Toco-Photoxil was efficiently taken up by the reticulo-endothelial system (RES) system. It was evident from the data that highest percentage of Toco-Photoxil was accumulated in the liver followed by spleen, lungs and heart (Supplementary Fig. S6a). Plasma was also analyzed for the presence of Toco-Photoxil which showed negligible presence.

Toco-Photoxil accumulation in liver on day 1 was found ~60% which is in good agreement to the already reported Herceptin-conjugated and doxorubicin-loaded half-shell Au PLGA nanoparticles^8^. However, in comparison to previous reports of targeted, drug-loaded and multifunctional Au PLGA nanoparticles, interestingly no accumulation of Toco-Photoxil in kidney was noted. Further, a very low quantity accumulation of Toco-Photoxil in spleen and lungs again emphasize the inert surface property and physiological stability of the material for superior homing capability and good renal clearance which is substantially fulfilled by Toco-Photoxil (Supplementary Fig. S6a).

To further determine the pharmacological kinetics of targeted and non-targeted Toco-Photoxil; as well specificity of folate targeting to major organs. Targeted and non-targeted Toco-Photoxil were injected systemically in equal amount, and percentage accumulation was determined in major organs at 24 and 72 h. At 24 h, the percentage accumulation of Toco-Photoxil and FA-Toco-Photoxil in liver was determined to be ~60% and ~90% respectively. At 72 h, the percentages of the same materials were reduced to ~40% and ~70% respectively. In spleen and other major organs also, the accumulation of Toco-Photoxil was lesser than FA-Toco-Photoxil and a similar pattern of degradation was observed. (Supplementary Fig. S7b).

### Histopathology, hematology, and serum biochemical analysis show good biocompatibility

Immune cells and plasma proteins (opsonins) could greatly reduce the dosage of nanoparticles at the targeted location due to hemolysis, thrombogenicity and complement activation^20^. Thus, hematology and full spectrum serum biochemical analysis were performed by harvesting the blood from mice prior sacrificing them on day 28 after Toco-Photoxil injection. It was observed that hematological parameters evaluated (i.e. hemoglobin, RBC, WBC, platelets, packed cell volume, neutrophils, eosinophils, lymphocytes, and monocytes) in test and control, did not show any significant difference (*P* > 0.05). Serum was analyzed for various biochemical parameters such as liver function indicator by serum glutamic oxaloacetic transaminase/aspartate amino transferase (SGOT/AST), serum glutamic pyruvic transaminase/alanine amino transferase (SGPT/ALT), alkaline phosphatase (ALKP), as well as total proteins, total albumin and total globulin was checked. While for nephrotoxicity, the level of blood urea nitrogen, creatinine, calcium, phosphorus and electrolytes- Na, K, Cl was determined. In addition, myocardial lesions, anemia, *etc*. was also checked by determining the level of creatinine phosphokinase and lactate dehydrogenase. In all cases no major alteration between the control and test was observed, which further assures the passiveness and non-toxicity of Toco-Photoxil (Supplementary Fig. S8a).

Further, one month after intravenous injection of Toco-Photoxil, all mice survived with no abnormal symptoms or behavior. There was also no change in general appearance and apparent loss in weight of organs harvested at the different time period (Supplementary Fig. S6b). Coefficient of major organs (liver, kidney, lungs and spleen) was also determined after one month of Toco-Photoxil administration, where no significant change was observed in comparison to control (Supplementary Fig. S6c). Even no erythema or edema was observed at the injection site. To determine the *in vivo* biocompatibility, histopathology of major organs was also performed which shows no apparent sign of overt microscopic pathological changes between the control and test organs (Supplementary Fig. S8b).

### Toco-Photoxil tuned at narrow absorbance shows superior photothermal transduction

**Figure 5:**
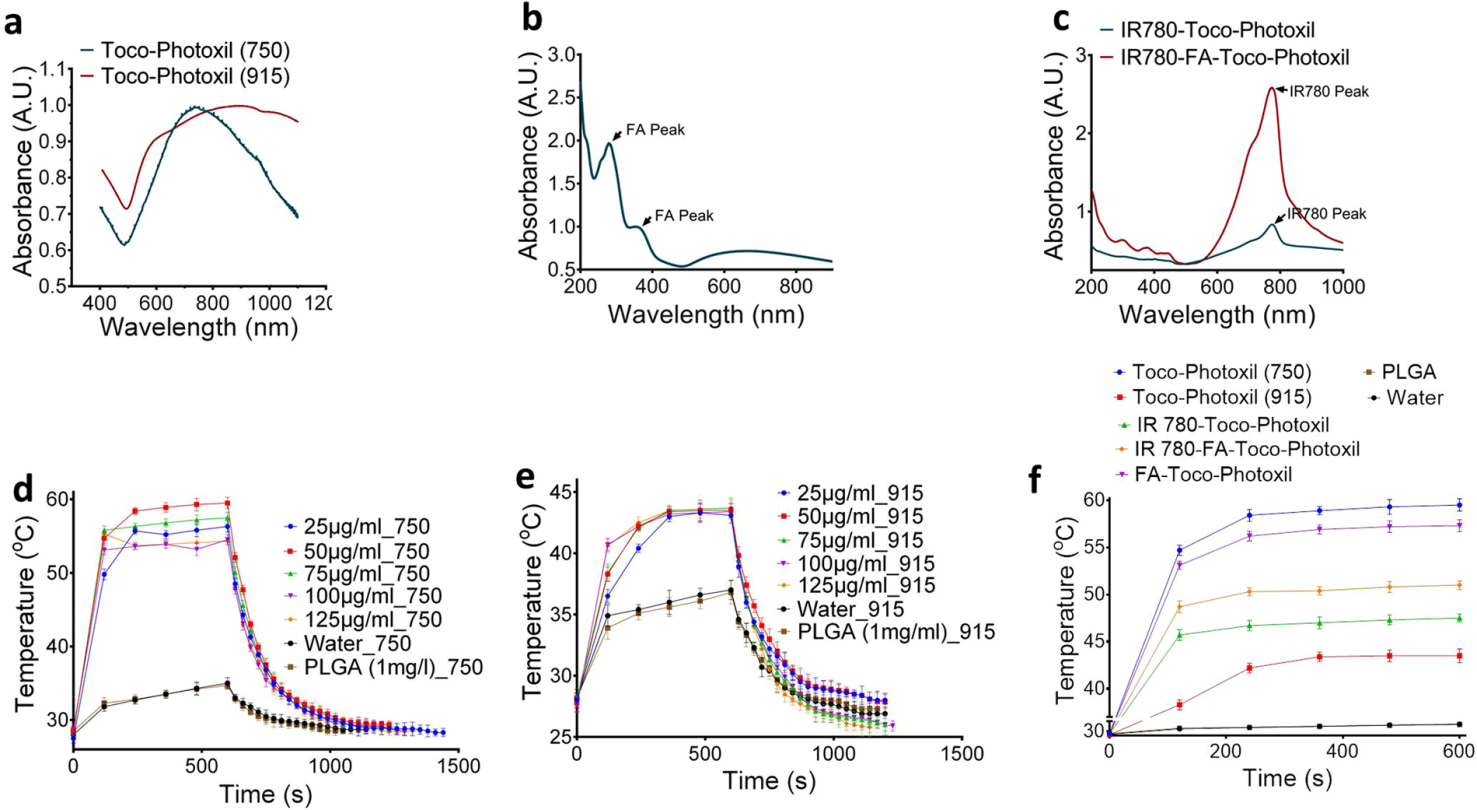
Absorbance and photothermal transduction efficiency. (**a**) Absorbance spectra of Toco-Photoxil tuned at 750 nm (narrow) and 915 nm (broad). (**b**) Absorbance spectra of FA-Toco-Photoxil showing peak due to FA and Toco-Photoxil. (**c**) Absorbance spectra of IR780 attached Toco-Photoxil and FA-Toco-Photoxil. (**d,e**) Photothermal transduction of Toco-Photoxil (750) & Toco-Photoxil (915) at various concentration and same low power setting of laser (~650 mW/cm^2^). (**f**) Temperature rise corresponding to laser irradiation on Toco-Photoxil (750), Toco-Photoxil (915), IR780-Toco-Photoxil, FA-Toco-Photoxil, IR780-FA-Toco-Photoxil, water, and PLGA NPs.

Photothermal transduction experiment was set to determine the increment in temperature due to NIR laser irradiation over Toco-Photoxil material. The Toco-Photoxil (50 µg/ml in 100 µl volume) tuned to an absorbance of 750 nm attained 53 ± 0.5 °C within 2 min. of irradiation and further attained the maximum temperature of 59 ± 0.3 °C within 5 min. of laser irradiation (650 mW/cm^2^). However, when the concentration raised up to 125 µg/ml, temperature recorded was little bit less than that attained at 50 µg/ml (Fig. 5d,f). Whereas, Toco-Photoxil tuned to broad absorbance having peak at 915 nm showed much lower increment in temperature and recorded a maximum of 42 ± 1 °C after 6 min. of laser irradiation (650 mW/cm^2^) even after the concentration was raised to 125 µg/ml (Fig. 5e,f). In control samples, i.e. in blank PLGA nanoparticle or Milli-Q containing tubes, the temperature did not exceed 38 °C while using either of the two laser sources (Fig. 5f).

Further, to determine the photothermal efficiency (*ƞ*) of 750 nm (narrow) and 915 nm (broad) tuned Toco-Photoxil the following equation was used^21^.

$${\rm{\eta }}=\frac{hS({T}_{Max}-{T}_{Surr})-{Q}_{Dis}}{I(1-{10}^{-{A}_{750,915}})}$$

Here, the heat transfer coefficient in the sample well surface area (*hS*) was found to be 8.4 mW and 9 mW for 750 nm and 915 nm tuned Toco-Photoxil respectively. The value of the steady-state maximum temperature (*T*_*Max*_) attained by the Toco-Photoxil subtracted from the ambient room temperature (*T*_*Surr*_) was determined as 21.7 °C and 14 °C for 750 nm and 915 nm tuned Toco-Photoxil respectively. Also, the energy input based on the amount of heat generated by only solvent (i.e. water) in sample well or the baseline energy input (*Q*_*Dis*_ = 39.55 mW), the laser power (*I* = 650 mW/cm^2^), and the absorbance of 750 nm and 915 nm tuned Toco-Photoxil (*A*_750, 915_ = 0.8497, 0.5321) were also determined. Thus, the calculated value of photothermal transduction efficiency (*ƞ*) of 750 nm and 915 nm Toco-Photoxil turned out to be 25.6% and 20.8% respectively, supporting our results as described above. Detailed calculation is provided in Supplementary Information and Supplementary Fig. S9.

### Importance of narrow absorbance for photothermal therapy in preclinical setting

**Figure 6:**
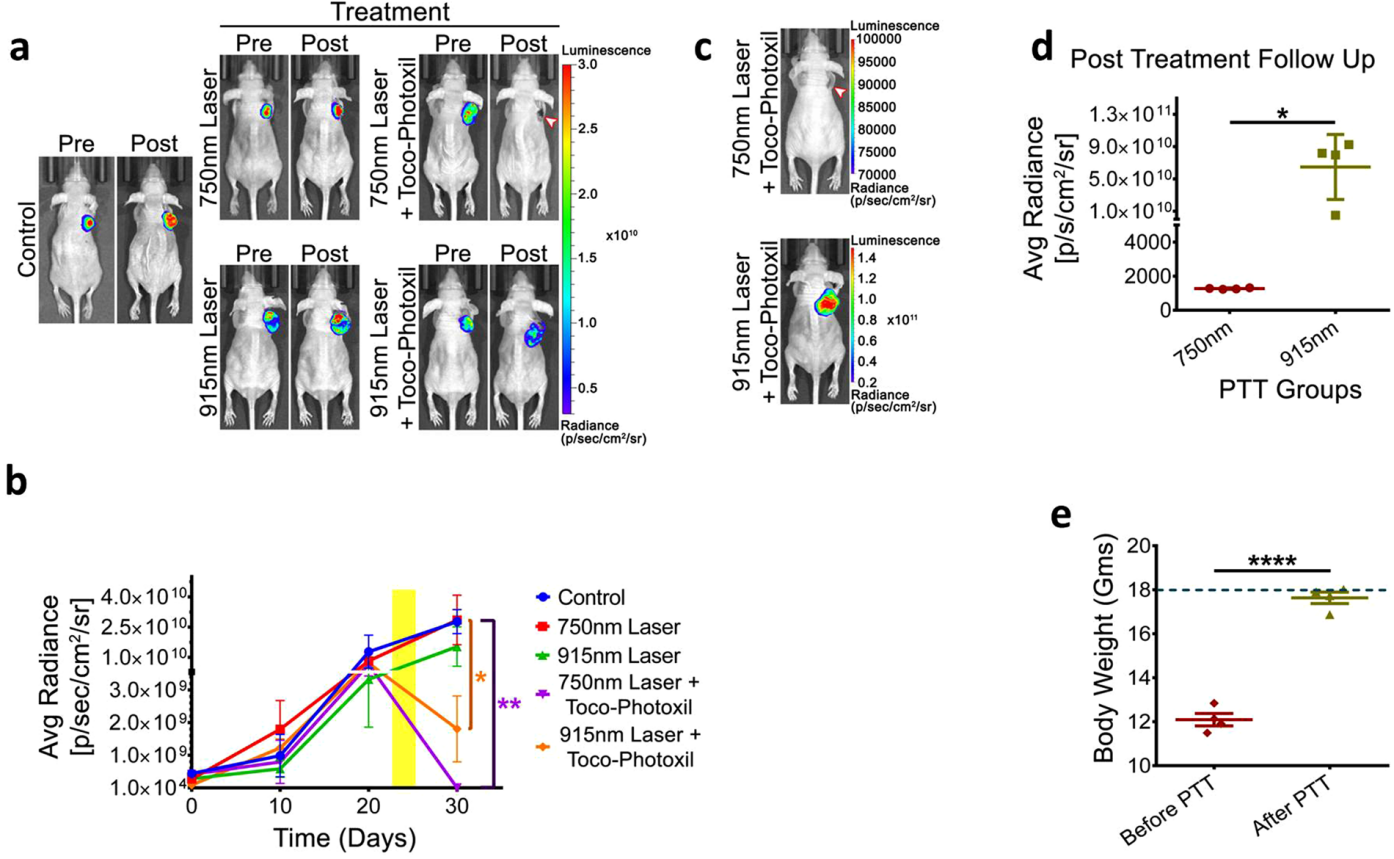
Comparative photothermal ablation of tumor following intratumoral injections of Toco-Photoxil tuned to 750 nm and 915 nm in HT1080-*fluc2-turboFP* xenograft model. (**a**) Representative pre- and post-photothermal treatment *in vivo* bioluminescence images of mice bearing HT1080-*fluc2-turboFP* tumor xenografts. (**b**) Quantitative assessment of bioluminescence signal to measure therapy response (yellow regions marks the photothermal treatment period) (*indicates *P* < 0.05 and ** indicates *P* < 0.01). (**c**) Representative bioluminescence images of post-treatment follow-up of mice treated with 750 nm and 915 nm tuned Toco-Photoxil and laser (arrow head indicates the treated tumor region). (**d**) Quantitative assessment of bioluminescence signal output 15 days post photothermal treatment (* indicates *P* < 0.05). (**e**) Graph represents change in body weight pre- and post-photothermal treatment (dotted line represents the average body weight of a non-tumor bearing BALB/c NUDE mice) (**** indicates *P* < 0.0001).

To further understand the therapeutic efficacy of Toco-Photoxil and setting up the relevance of narrow and broad absorbance peak for photothermal therapy when performed in physiological setting, *in vivo* photothermal therapy was carried out using the HT1080-*fluc2-turboFP* xenograft model in BALB/c NUDE mice. After random segregation of mice (n = 4 per group) with well grown tumors, 750 nm tuned and 915 nm tuned Toco-Photoxil were administered in respective groups *via* intratumoral injection on day 24. 750 nm laser irradiation was performed on groups II and IV, while 915 nm laser irradiation was performed on group III and V tumors twice for 4 min. duration each on successive days (day 24 and 25). All the animals were imaged by injecting D-luciferin bioluminescence substrate as a measure of viable tumor mass till the termination of the experiment. The study reveals a drastic drop in bioluminescence signal (1.94 × 10^5^ ± 1.25 × 10^5^ p/sec/cm^2^/sr) on the 30^th^ day in 750 nm laser-treated animals when compared to untreated control counterpart (2.77 × 10^10^ ± 5.95 × 10^9^ p/sec/cm^2^/sr), confirming massive ablation of tumor mass (*P* = 0.0097). Although, a log-fold reduction in the bioluminescence signal was also observed in the 915 nm laser treated group with the average radiance of 1.805 × 10^9^ ± 1.009 × 10^9^ p/sec/cm^2^/sr (*P* = 0.0128), indicates lesser therapeutic efficacy achieved within the same duration (Fig. 6a). Additionally, the incomplete regression of tumor mass after photothermal therapy in group V resulted in quick relapse within 15 days as confirmed by bioluminescence imaging (*P* = 0.0183), whereas the group IV animals showed no signs of recurrence during the month long of follow-up period (Fig. 6b). Furthermore, the ablation of tumor burden in group IV animals also correlates well with significant recovery in body weight from 12.09 ± 0.28 g to 17.63 ± 0.26 g (*P* < 0.0001), suggesting an improvement in their overall survival condition during the post-PTT period (Fig. 6c).

### Toco-Photoxil mediated PTT is universal across cancer cell types

As the therapeutic action of photothermal therapy is physical in nature, the credibility of photothermal damage by Toco-Photoxil was estimated in 5 widely different cancer cell types in culture setting, i.e. MCF-7 (breast adenocarcinoma), MDA-MB-231 (basal-like invasive breast cancer), SKBR-3 (invasive ductal breast carcinoma), HeLa (cervical cancer), and HT1080 (human fibrosarcoma) cells. All cells were incubated for about 4 h with 750 nm tuned Toco-Photoxil at a concentration of ~50 µg/ml per 100 µl prior to the laser irradiation and then irradiated using a 750 nm (650 mW/cm^2^) NIR laser for 4 min. continuous beam exposure. The cells treated with Toco-Photoxil alone or laser alone exhibited greater than 90% cell viability, while a steep drop (~20% viable cells remaining) was observed in the case of combined (Laser + Toco-Photoxil) treatment (*P* < 0.0001) (Supplementary Fig. S10a). For the purpose of quantitative assessment of cells undergoing apoptosis, cells were also stained with propidium iodide (PI) after laser irradiation in each well and assessed using fluorescence microscope. MCF-7 cells stained with PI (red) remained nearly the same as in control cells or the cell treated with either Toco-Photoxil or laser alone. However, a dramatic increase in the number of PI-stained cells was seen in cells treated with Toco-Photoxil and laser combination (Supplementary Fig. S11). To further corroborate the extent of photothermal damage as well as to determine the mechanism of cell death, flow analysis was performed in MCF-7. Nearly 80% cell death observed in flow result was concordant to our findings in MTT assay (Supplementary Fig. S10b). In forward and side scatter graph, it was observed that after PTT, population showed shift in both FSC-A and SSC-A i.e. reduction in size and increase in granularity respectively, which further validate the apoptotic nature of the cells’ death after photothermal treatment at mild hyperthermia^22,23^ (Supplementary Fig. S10c).

### Percentage tumor bioavailability in response to surface modification for targeting folate receptors

**Figure 7:**
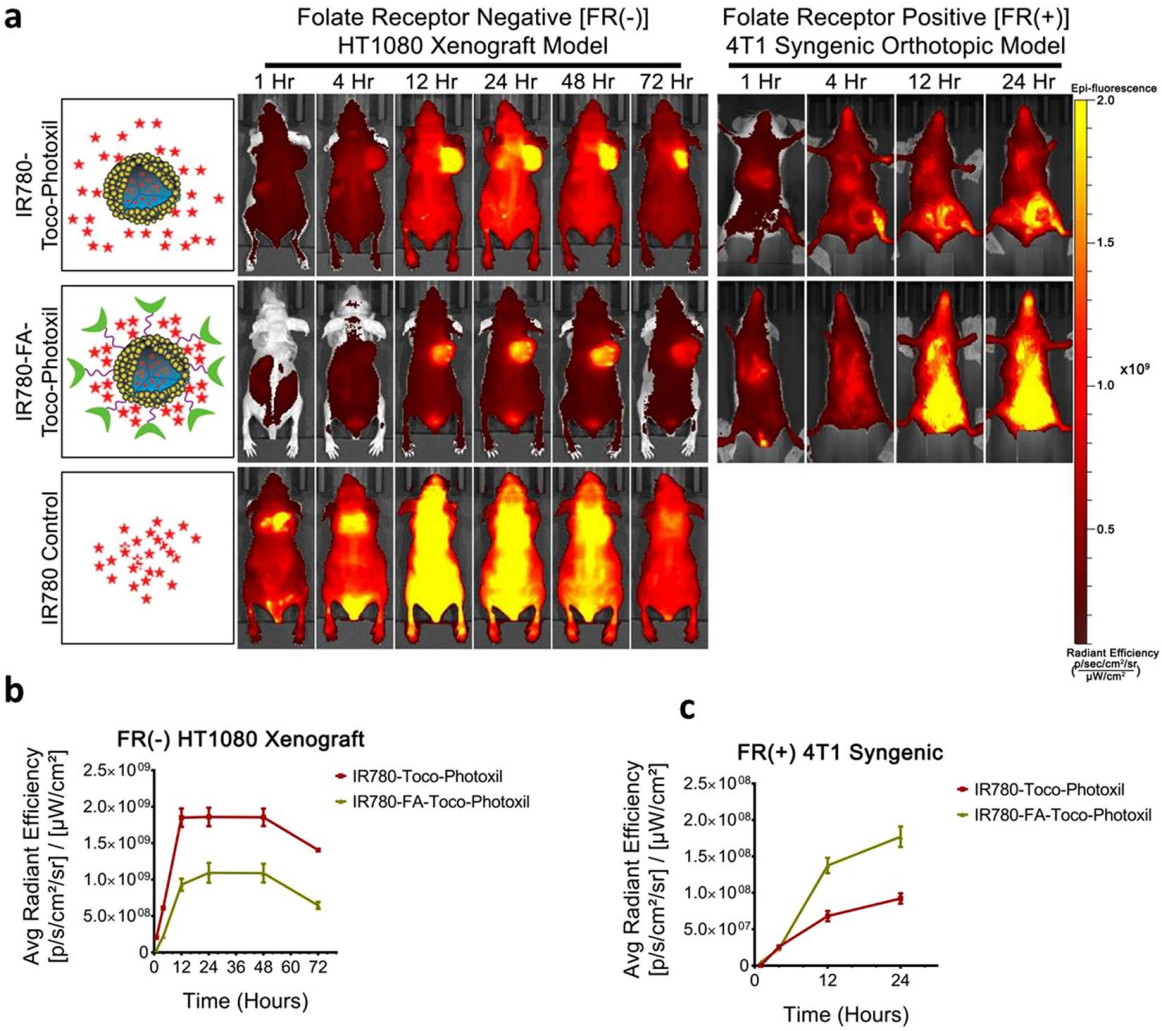
Near Infra-Red Fluorescence (NIRF) imaging of IR780 dye tagged Toco-Photoxil and FA-Toco-Photoxil after systemic delivery in HT1080 FR(−) xenograft and 4T1 FR(+) orthotopic tumor-bearing mice. (**a**) Representative NIRF images of HT1080 xenograft and 4T1 syngeneic tumor bearing mice at different time points after systemic delivery of IR780-Toco-Photoxil (top), IR780-FA-Toco-Photoxil (middle), and IR780 dye control (bottom). (**b**) Quantitative assessment of fluorescence signal in HT1080 tumors of mice injected with IR780-Toco-Photoxil, and IR780-FA-Toco-Photoxil. (**c**) Quantitative assessment of fluorescence signal in 4T1 tumors of mice injected with IR780-Toco-Photoxil, and IR780-FA-Toco-Photoxil.

To understand the targeting as well as its tumor homing capability in response to surface modification and folate receptors, Toco-Photoxil tagged with NIR dye IR780 was developed and introduced systematically. IR780-Toco-Photoxil started accumulating in HT1080-*fluc2-turboFP* tumor within 4 h [average radiant efficiency 6.11 × 10^8^ ± 2 × 10^7^ (p/sec/cm^2^/sr)/(µW/cm^2^)] and reached near saturation within 12 h [1.85 × 10^9^ ± 9 × 10^7^ (p/sec/cm^2^/sr)/(µW/cm^2^)]. Intermittent imaging of the same animals on a daily basis thereafter reveals that strongest signal lasts till 48 h [1.86 × 10^9^ ± 8.5 × 10^7^ (p/sec/cm^2^/sr)/(µW/cm^2^)]. Further, a comparison was also made by developing IR780-FA-Toco-Photoxil which can actively target the folate receptor positive tumors. It showed marked improvement in lowering the background signal in comparison to IR780-Toco-Photoxil (Fig. 7). The maximum enrichment of IR780-FA-Toco-Photoxil was observed at 24 h [1.09 × 10^9^ ± 6.8 × 10^7^ (p/sec/cm^2^/sr)/(µW/cm^2^)] with less leaching of dye to other body parts. In the control mice injected with IR780 dye alone, fast accumulation of IR780 in tumor site was noticed (started enriching in tumor within the first hour) and reached to the peak within 4 h, but thereafter started diffusing out from the tumor bed leaving almost very less tumor specific fluorescence at 72 h time point (Fig. 7a,b).

**Figure 8:**
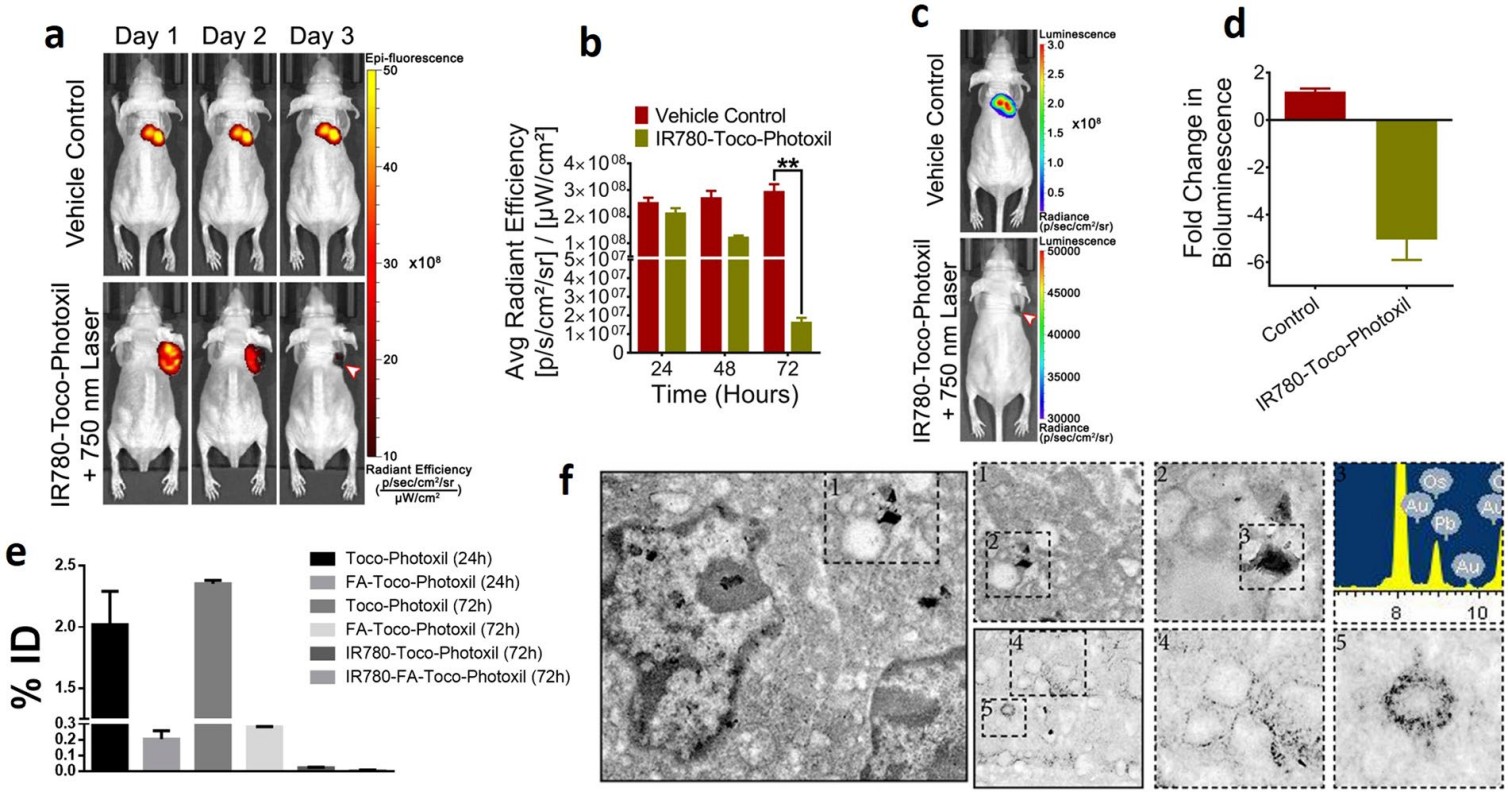
Photothermal therapy after systemic delivery of Toco-Photoxil and percentage accumulation in tumor in comparison to other surface modified Toco-Photoxil. (**a**) Qualitative representation of TurboFP fluorescence images of mice bearing HT1080*-fluc2-turboFP* tumors during the course of photothermal treatment (arrow head indicates the treated tumor region). (**b**) Quantitative assessment of changes in light output of the TurboFP fluorescent protein (*P* < 0.01). (**c**) Representative follow up bioluminescence images of mice at day 10 (arrow head indicates the treated tumor region). (**d**) Fold change in bioluminescence light output between the vehicle control treated mice and mice treated with a combination of Toco-Photoxil and 750 nm laser. (**e**) Percentage tumor uptake of Toco-Photoxil, FA-Toco-Photoxil, IR780-Toco-Photoxil, and IR780-FA-Toco-Photoxil (**** indicates *P* < 0.0001). (**f**) FEG-TEM images of 4T1 tumor section showing accumulation of Toco-Photoxil.

By experiencing a sustained and high EPR mediated accumulation of IR780-Toco-Photoxil, photothermal therapy after systemic delivery of the nanoshells was commenced by localized laser irradiation between 24 and 48 h time points, which resulted in total ablation of the solid tumor mass. Tumor location was further cross-validated by performing both TurboFP fluorescence and firefly luciferase bioluminescence reporter imaging procedures. At post-PTT end point (72 h), the TurboFP fluorescence signal of the HT1080-*fluc2-turboFP* tumor in the IR780-Toco-Phtoxil treated group [1.7 × 10^7^ ± 2.1 × 10^6^ (p/sec/cm^2^/sr)/(µW/cm^2^)], which was significantly lower than the IR780 dye control group [3 × 10^8^ ± 2.6 × 10^8^ (p/sec/cm^2^/sr)/(µW/cm^2^)] (*P* = 0.0083) represented in Fig. 8a,b. *In vivo* non-invasive bioluminescence imaging was further carried out on day 10 (although IR780-Toco-Photoxil mice survived more than 4 months) post-photothermal treatment to confirm any sign of tumor cell revival at the site (Fig. 8c). The animals from IR780-Toco-Photoxil group exhibited a near 5-fold lower bioluminescence output as compared to the IR780 dye control group (Fig. 8d) indicating a negligible revival of the HT1080 tumor.

Thereafter, the same study was also performed in FR(+) orthotopic tumor model, using mouse breast cancer cell line 4T1. Contrary to the FR(−) HT1080 xenograft model, NIRF imaging reveals that higher amount of IR780-FA-Toco-Photoxil particle accumulates in 4T1 orthotopic tumor in comparison to IR780-Toco-Photoxil (Fig. 7a,c). The results were concordant with the comparative uptake study of FA-Toco-Photoxil performed in HT1080 and 4T1 cells (Supplementary Fig. S7a). However, when results were validated with ICP-MS, it was found that nanoparticle accumulation was much higher in case of IR780-Toco-Photoxil (0.025% ID) than the IR780-FA-Toco-Photoxil (0.005% ID), while the highest was observed in bare Toco-Photoxil (2.3% ID) irrespective of the tumor models. The enrichment of Toco-Photoxil increased from 2.0 to 2.3% within 72 h after injection while the increment in FA-Toco-Photoxil accumulation was very less and hardly it was~0.3% even after 72 h of intravenous injection (Fig. 8e,f).

### Toco-Photoxil supports X-ray imaging

Gold nanoparticles have been described as efficient diagnostic contrast agent due to its higher X-ray attenuation power than iodine and thus providing longer imaging time because of delayed renal clearance with minimal toxicities^24^. Toco-Photoxil was scanned for X-ray imaging and was found that even at four times less concentration, X-ray images showed comparable contrast to the commercially used iodine-based contrast agent e.g. Omnipaque (Supplementary Fig. S12a,b).

4-D X-ray imaging (FDXM) was performed with the help of phantom study based on 1% agarose gel matrix (because of its less attenuation to the electron beam and its potential to easily form a porous bulk like structure). It was observed that Toco-Photoxil embedded phantoms provided much better contrast and topological features than the plain agarose phantom as after image processing we were also able to see the internal anatomy of phantoms. (Supplementary Fig. S12c and Supplementary Movie).

## Discussion

There are various anticancer nanomedicines, but so far only a handful of them have cleared the preclinical and clinical trial phases prominently. Some of them have shown promising results in the preclinical trial, but failed to prove efficacy in clinical phases either due to toxicity, poor efficacy, or heterogeneous nature of tumor in clinical setting^15,25^. The initial clinical trial of Doxil in patients suffering from broad types of cancer demonstrated a promising tumor specific accumulation in comparison to the adjacent normal tissues^26,27^. Similarly, while designing this product, our main aim was to ensure maximum accumulation of particles within the tumor to provide a promising stand-alone PTT to ensure significant tumor regression without additional chemo drugs in combination. Our results have clearly demonstrated that Vitamin E modified Toco-Photoxil is a highly inert and stable material at physiological pH and temperature that ensures sufficient accumulation by EPR after systemic delivery of the material in at least the two types of solid tumors tested. At this point, we speculate that this high yield, disintegrable, metabolically inert, and low cost, quick therapy procedure obtained by using Toco-Photoxil would prove beneficial to treat a variety of the solid tumors located at low tissue depths.

The very low or no *in vitro* cytotoxicity of Toco-Photoxil could be attributed to several factors including no drug involvement, inherent non-reactive metallic gold nanoparticles, highly biocompatible PLGA core as well as glycol chitosan and TPGS material used. The similarity of oxidative index of Toco-Photoxil to FA-(GSH)-Toco-Photoxil without the requirement of antioxidant moiety could also be attributed to involvement of TPGS during nano-formulation. Until now none of the published literature related to drug loaded multifunctional gold-PLGA hybrid nanomaterials have investigated the possibility of hemolysis. In comparison to earlier charted drug-loaded multifunctional nanoparticles having potential for hemolysis due to its immune (drug-specific antibody) and non-immune mediated (drug-RBCs membrane) interactions, Toco-Photoxil don’t show any hemolysis and clearly fulfills the criteria of intravenous nanomaterials as per ISO 746. The energy released due to binding of Toco-Photoxil with RBC membrane also seems to be lesser than that required for bending of RBC membrane around the Toco-Photoxil. It evidences the inert nature of Toco-Photoxil in terms of geometry and surface chemistry. Based on the thorough and controlled hemolysis analysis performed, no significant effect before and after incubation with the Toco-Photoxil clearly suggests non-adsorbent and safe nature of the material.

There was no major evidence of biochemical or histopathological toxicity recorded even after using more than double the therapeutic amount needed. It further reflects the passive, non-toxic nature of Toco-Photoxil *in vivo* as well as in major organs. In comparison to our previous study^12^, where 48% of LiposAu accumulated in liver and due to thermos-labile lipid core used, faster disintegration of material helped in clearing 97% of the injected dose within 7 days. Here, 60% of Toco-Photoxil accumulates in liver within a day, and disintegration rate is slower because of the stable nature of PLGA polymer. Although, continuous elimination of gold through urine and no retention in kidney entails its slower but successful renal clearance over 4 weeks’ time course measured. Together, these systematic characterizations of Toco-Photoxil provide a vivid insight over the rationale design used for manufacturing this theranostic nanomaterial and thus establish a strong basis for testing its photothermal potential.

In *in vitro* photothermal characterization, there was no dose dependent effect observed because of temperature saturation obtained even at the lowest concentration. Increased material concentration leads to more scattering than absorbance. In case of 915 nm broad tuned Toco-Photoxil, very little increment in temperature with increased concentration establishes that narrow absorbance is critically required for achieving the hyperthermic temperature. The FA and IR780 functionalization further decreased photothermal transduction potential, probably because of the disturbance of plasmon resonance and reduced stability leading to aggregation. The *in vitro* findings were further validated in preclinical setting, wherein, the realization for *in vivo* hyperthermic potential to ablate cancer cells provide critical insight that narrow absorbance in NIR range is of prime importance. It helps in efficient rise in critical temperature (>42 °C) when PTT performed in physiological setting using the biological acceptable low power (~650 mW/cm^2^).

In NIRF imaging directed therapy, efficient accumulation of IR780-Toco-Photoxil with high signal to background ratio (perhaps due to efficient entrapment of dye by the FA-GSH corona over the nanoshells) helped in retarding the growth of tumor by repeated laser irradiation at 24 and 48 h. However, the same treatment procedure in the case of IR780-FA-Toco-Photoxil wasn’t sufficient to completely remove the tumor. It is worth to note here that IR780 dye preferably accumulates in an energy-dependent manner inside the mitochondria of tumor cells and cause apoptosis by increasing the ROS production and depolarization of the mitochondrial membrane^28^. However, applicability of IR780 is still limited due to its inability to dissolve in most commonly accepted pharmaceutical solvents as well as its concentration dependent toxicity. To overcome this problem, IR780 has been encapsulated inside the nanocarriers^29^. Also, it is well evidenced in literature that loading of hydrophobic drug and ligand conjugation affects the property of nanoparticles like size, zeta potential, photothermal efficiency etc. unless they are encapsulated or conjugated adeptly^30,31^. Discordance of ICP-MS and NIRF measurements observed in FR + ve 4T1 tumor might be due to this same fact. Although, we have no conclusive experimental evidence at this point, IR780-FA moiety detachment from the Toco-Photoxil in the acidic environment of tumor bed might be another reason.

Computed Tomography (CT) has revolutionized the field of radiological imaging by providing 3D tomographic information. However, as accuracy outcome of X-ray and CT imaging in patients with tumor in thorax and abdomen region is often compromised due to breathing, cardiac and gastrointestinal tract movement, a precise delineation is required to spare normal tissues from the tumor tissue. Toward this, various techniques, such as 4-D CT or respiration-co-related CT are under development to compensate such artifacts and therefore these techniques are found to be viable and clinically effective tool for assessing the exact tumor mass, position and shape during the patient respiration cycle. Toco-Photoxil embedded phantoms shows comparative contrast to the iodine based contrast agent Omnipaque at 5 times less concentration due to high X-ray attenuation power. The further image processing helped in visualizing the internal anatomy of the phantom as well as distribution of Toco-Photoxil. At this point, we envisage that it will help in imaging directed therapy and thus easily modulating the dosage of therapeutic agent as per the patient’s health recovery.

In summary, using seed-mediated *ex-situ* method of gold coating has helped in providing better stability responsible for long circulation life within body and ultimately helped in enhanced EPR mediated accumulation from systemic delivery. In comparison to our earlier product, LiposAu, which showed almost no EPR mediated accumulation, Toco-Photoxil shows the clear sign of success overcoming the barrier of intra-tumoral material delivery dependence^12^. Further, to prevent accumulation of metallic gold nanoparticles in the body, which may be seen as a legitimate health concern for clinical application, we have ensured disintegration especially in *in vivo* setting and thus maximum clearance of the metal particles within a short period. Based on the evidences gathered during this study, the space between the gold seeds seems to be more pronounced after photothermal therapy, and thus allowing the hydrolytic enzymes to act on the polymer core which further underpins the disintegration ability of Toco-Photoxil. With detailed calculations of the photothermal conversion efficiency of the materials developed during this study, striking results clearly demonstrate the relevance of narrow range NIR absorbance for efficient PTT in a 3D setting.

Thus, application of such a simple form of nano-sized, hybrid material holds great promise towards the long stride of effective elimination or containment of solid tumor mass without causing any potential side effects.

## Electronic supplementary material

Supplementary Information

Movie_FDXM
