# Supplementary material for: Enhanced EPR directed and Imaging guided Photothermal Therapy using Vitamin E Modified Toco-Photoxil
Source: Sci Rep. 2018 Nov 12;8:16673. doi: 10.1038/s41598-018-34898-3 (PMC6232175; doi:10.1038/s41598-018-34898-3)
Supplement: Supplementary file 1 — Supplementary Information [file 41598_2018_34898_MOESM1_ESM.docx]

**SUPPLEMENTARY INFORMATION**

**Enhanced EPR directed and Imaging guided Photothermal Therapy using Vitamin E Modified Toco-Photoxil**

Deepak S. Chauhan^a§^, Amirali B. Bukhari^b§^, Gayathri Ravichandran^b^, Ramkrishn Gupta^a^, Liya George^a^, Radhika Poojari^a^, Aravind Ingle^c^, Aravind K. Rengan^d^, Asifkhan Shanavas^e^, Rohit Srivastava^a*^, and Abhijit De^b*^

^a^Department of Biosciences and Bioengineering, Indian Institute of Technology Bombay, Powai, Mumbai, India

^b^Molecular Functional Imaging Lab, Advanced Centre for Treatment, Research and Education in Cancer (ACTREC), Tata Memorial Centre, Kharghar, Navi Mumbai, India

^c^Laboratory Animal Facility, Advanced Centre for Treatment, Research and Education in Cancer (ACTREC), Tata Memorial Centre, Kharghar, Navi Mumbai, India

^d^Department of Biomedical Engineering, Indian Institute of Technology Hyderabad, India

^e^Institute of Nano Science and Technology, Mohali, Punjab, India

^§^These authors contributed equally

^*^**Corresponding Author**

Dr. Abhijit De

Scientific Officer ‘F’

Molecular Functional Imaging Laboratory

ACTREC, Tata Memorial Centre

Kharghar, Navi Mumbai – 410210, India

Phone: +91-22-2740 5038

E-mail: [ade@actrec.gov.in](mailto:ade@actrec.gov.in)

^*^**Co-corresponding Author**

Dr. Rohit Srivastava

Professor

Department of Biosciences and Bioengineering

Indian Institute of Technology Bombay

Powai, Mumbai – 400076, India

Phone: +91-22-2576 7746

Fax: +91-22-2572 3480

E-mail: [rsrivasta@iitb.ac.in](mailto:rsrivasta@iitb.ac.in)

**Materials and methods**

**Materials**

Poly (DL-lactide-co-glycolide) (PLGA; L: G molar ratio: 50:50, MW: 17000 Da, PURAC Biomaterials, The Netherlands). Polyvinyl alcohol (PVA; MW: 17000-23000 Da), d-α tocopheryl polyethylene glycol 1000 succinate (TPGS), chitosan (CHT; low molecular weight), glycol chitosan (GCHT; ≥60% (titration), 3-(4,5-dimethylthiazol-2-yl)-2,5-diphenyl tetrazolium bromide (MTT), propidium iodide (PI) and IR-780 dye have been procured from Sigma-Aldrich, USA. Chloroauric acid (HAuCl_4_) and N-(3-dimethylaminopropyl)-N′-ethylcarbodiimide hydrochloride (EDC) was procured from Spectrochem, India. Potassium carbonate (K_2_CO_3_) and sodium bicarbonate (NaHCO_3_) were procured from Merck, India. Tetrakis (hydroxymethyl) phosphonium chloride (THPC 80%) was procured from Acros Organics, Belgium. Hydroxylamine hydrochloride (HACl) was procured from Fisher Scientific, USA. Dulbecco’s modified Eagle’s medium (DMEM), Roswell Park Memorial Institute (RPMI-1640), McCoy's 5A medium, fetal bovine serum (FBS), trypsin-EDTA, phosphate buffered saline (PBS), antibiotic-antimycotic solution and dialysis membranes were procured from HiMedia Laboratories, Mumbai, India. D-luciferin potassium salt was purchased from Biosynth, Switzerland. Glutathione reduced (GSH) from Sisco Research Laboratories, India Pvt. Ltd. N-Hydroxysuccinimide (NHS) from Acros organics, Belgium.

**Synthesis of Toco-Photoxil**

Toco-Photoxil and FA-Toco-Photoxil was synthesized as following:

1. **Preparation of GCHT-PLGA nanoparticles**

Glycol chitosan coated PLGA nanoparticles (GCHT-PLGA NPs) were prepared using emulsion-solvent evaporation method.^1^ Glycol chitosan (0.025 % w/v) was dissolved in 20 ml of Milli-Q water at room temperature containing TPGS (0.02 % w/v). 25 mg PLGA was added to 10 ml acetonitrile and kept overnight for dissolution. Next day, the organic phase was added dropwise into an aqueous phase at a speed of 100 µl/min under vigorous stirring (~1300 rpm) at room temperature. The emulsion was sonicated for 10 min. with the pulse of 5 sec. ON and 2 sec. OFF using probe sonicator (SONICS Vibra-Cell, USA). The solvent was evaporated for 36 h at room temperature. GCHT-PLGA NPs were washed thrice at 15000 rpm for 1 h. GCHT-PLGA nanoparticles were characterized using dynamic light scattering (DLS, Brookhaven Instruments, USA), Zeta Potentiometer (Brookhaven Instruments, USA), field emission gun-scanning electron microscope (FEG-SEM, JEOL, Japan), Fourier transform infrared spectroscopy (FTIR, 3000 Hyperion Microscope with Vertex 80 FTIR System, Bruker, Germany).

1. **Preparation of gold seeds**

The reduction of chloroauric acid to gold seeds using THPC was performed as previously described by Pham et al.^2^ Briefly, 9 ml of Milli-Q water, 100 µl of 1 M NaOH and 200 µl of THPC solution (1 ml Milli-Q water containing 12 µl of 80% THPC) were added. The solution was stirred for 5 min. After that, 400 µl of 5 mM chloroauric acid was added. The appearance of dark brown color indicated the gold seeds (3 - 4 nm) formation. Gold seeds formed were characterized using transmission electron microscope (TEM, Philips CM200, Amsterdam) and field emission gun-transmission electron microscope (FEG-TEM, JEOL, Japan).

1. **Attachment of gold seeds to GCHT-PLGA NPs**

To attach the gold seeds on amine group of glycol chitosan coated PLGA nanoparticle, 4 ml of GCHT-PLGA (1 mg/ml) was added to 9 ml of gold seeds under vigorous stirring. The reaction mixture was incubated for 24 h in the dark. Next day, it was washed thrice at 4000 rpm for 1 h using Hitachi CF 15 RX II centrifuge to remove excess gold seeds. The light brown pellet was re-dispersed in 500 µl water. Attachment of gold seeds was confirmed by FEG-TEM.

1. **Shell growth**

THPC-gold seeds attached to glycol chitosan coated PLGA was used to form gold shells as previously described by Graf et al.^3^ and Pham et al.^2^ Gold hydroxide solution was prepared as previously described by Duff et al.^4^ Briefly, 10.8 mg of potassium carbonate (K_2_CO_3_) was dissolved in 40 ml of Milli-Q water for 10 min. After that, 3.2 ml of 5 mM HAuCl_4_ was added to the solution. After 5 min., color changed from yellow to colorless thereby indicating gold hydroxide formation. Gold hydroxide was aged for 24 h in the dark before use. Gold seeds attached GCHT-PLGA nanoparticles were added to the gold hydroxide solution. To this vigorously stirred mixture, 5 ml freshly prepared 1.87 mM hydroxylamine hydrochloride solution was added dropwise which gave a black precipitate. Gold coated Vitamin E modified GCHT-PLGA nanoparticles (i.e. Toco-Photoxil) were pelleted by centrifuging at 5000 rpm using Hitachi CF 15 RX II centrifuge and re-dispersed in Milli-Q water. Toco-Photoxil was characterized using the FEG-TEM, FEG-SEM, DLS, Zeta potentiometer. Also, the surface of Toco-Photoxil was analyzed with the help of X-ray photoelectron spectroscopy (XPS, AXIS Supra, UK with Al K- α source (225 W) Kratos Analytical).

1. **Synthesis and conjugation of folic acid-glutathione moiety**

In 5 ml of folic acid (FA, 25 mg in 5 ml of NaHCO_3_ buffer (pH ~ 6.5), 200 µl of N-ethyl-N′-(3-(dimethylamino) propyl) carbodiimide (EDC, 6.9 mg in 1 ml of water) and N-hydroxysuccinimide (NHS, 3.91 mg in 1ml water) were added. After 15 min., of activation, 1ml of glutathione (GSH, 1.382 mg in 2 ml of NaHCO_3_ buffer) was added to the activated FA prior adjusting pH ~ 8 (pH was adjusted with highly conc. Na_2_CO_3_ buffer). After 2.5 h of stirring, 5 ml of 150 µg/ml of Toco-Photoxil was added and incubated for another 6 h over stirring. Next day, the solution was washed 3 times, 30 min. each at 7000 rpm. FA conjugation was determined using 1H NMR (Varian Mercury Plus 300MHz NMR SPECTROMETER using D_2_O_2_ as a solvent), and UV-Vis spectroscopy.

***In vitro* assessment of Toco-Photoxil biocompatibility**

1 x 10^4^ L929 and NIH3T3 cells (procured from NCCS Pune, India) were seeded in a 96-well plate in triplicates. To determine the cytotoxicity of Toco-Photoxil, 200 µl of varying concentrations of Toco-Photoxil dispersed in media ranging from 25 µg/ml to 125 µg/ml were added and incubated for 24 h. Next day, the media was aspirated, and wells were washed off with PBS. Following this, 200 µl of fresh media containing 10 % MTT solution (0.5 mg/ml) was added and allowed to incubate for another 4 h at 37 °C. Media was then removed, and the blue formazan crystals were dissolved in 200 µl DMSO. Calorimetric measurements were done at an absorbance of 560 nm and 670 nm using a microplate reader (Tecan Infinite 200 PRO). The data was expressed in terms of percentage viability in comparison to the control group.

L929 cells were seeded in 6 well plate at a density of 1 x 10^4^ cells per well. After overnight culturing of cells, 200 µl of 125 µg/ml Toco-Photoxil and FA-Toco-Photoxil were added and left for 24 h. Cells were washed with PBS and stained with 10 µM of  (5-(and-6)-chloromethyl-2′,7′-dichlorodihydrofluorescein diacetate, acetyl ester) (CM-H2DCFDA). After 30 min. of incubation at 37 ^o^C, cells were repeatedly washed with PBS to remove the excess dye and finally dispersed in 500 µl of PBS. Reactive oxygen species (ROS) was measured using BD FACSVerse Flow Cytometer at Ex = 488 and signal were collected using 530 nm band pass filter. Data were analyzed using FlowJo 10. Cells incubated with H_2_O_2_ (50 µM for 30 min), CM-H2DCFDA stained untreated, and unstained untreated cells were used as controls. Each measurement was made on the fluorescence intensity of around ~10,000 cells and gates were set according to forward and side scattering to keep out the cell debris and cell doublets from the analysis.

**Hemolysis study**

Hemolysis assay was performed according to ISO/TR 7406. EDTA stabilized mice blood was obtained from Advanced Centre for Treatment, Research and Education in Cancer (ACTREC), India. 100 µl of blood was dispersed in 900 µl of PBS and repeatedly washed at 1000 rpm for 5 min. at least 5 times until the clear supernatant was obtained. Pelleted down RBCs were dispersed in 1 ml of PBS and stored in 4 ^o^C till use. 150 µl of RBC dispersed in PBS was added to 750 µl (125 µg/ml and 50 µg/ml) of Toco-Photoxil (pre-saturated in PBS for 1 h) and the mixture was incubated for 1 h and 24 h study at 37 ^o^C. After incubation, samples were pelleted down at 15000 rpm, and the absorbance of supernatant containing hemoglobin was recorded using TECAN Pro plate reader at 575 and 655 nm (reference wavelength). 750 µl of water and PBS were used as positive and negative control.

For SEM imaging, all procedure was same as explained above except after incubation, samples were pelleted down at 2000 rpm and dispersed in 200 µl of PBS. One drop of sample was added to 500 µl of 2.5 % of glutaraldehyde and kept at 4 ^o^C for 24h fixation. It was washed two times with 100 µl of water, and a drop was cast over an aluminum foil covered copper stubs.

**Photothermal transduction experiment**

During the photothermal transduction experiment, the heating and cooling temperature was also recorded at different time intervals, and photothermal efficiency (ƞ) was determined by the following equation.^5^

$$ƞ=\frac{\mathrm{hS}\left( T_{\mathrm{Max}}-T_{\mathrm{Surr}} \right)-Q_{\mathrm{Dis}}}{I(1-{10}^{-A_{750, 915}})}$$

Where h is the heat transfer coefficient, S is the sample well surface area, T_Max_ is the steady-state maximum temperature attained by Toco-Photoxil, T_Surr_ is the ambient room temperature, Q_Dis_ is the energy input based on the heat generated by the solvent and sample well or baseline energy input, I is the laser power, and A_750,915_ is the absorbance of 750 nm and 915 nm tuned Toco-Photoxil.

**Calculation to determine photothermal conversion efficiency**

According to a report by Roper *et al.*^5^, the total energy balance for the system can be expressed by equation (1)

$\sum_{i} m_{i}C_{p,i}\frac{dT}{dt}=Q_{NS}+Q_{Dis}-Q_{Surr}$ (1)

Where *m* and *C_p_* are the mass and heat capacity of water respectively, *T* is the solution temperature, *Q_NS_* is the energy input by Toco-Photoxil, *Q_Dis_* is the baseline energy input by the sample cell, and *Q_Surr_* is heat conduction away from the system surface by air.

The laser-induced source term, *Q_NS_* represents heat dissipated by electron-phonon relaxation of the plasmon of the Toco-Photoxil’ surface under irradiation by 750 nm and 915 nm laser:

$Q_{NS}=I\left( 1-{10}^{-A_{750,915}} \right)ƞ$ (2)

where *I* is incident laser power, *ƞ* is the conversion efficiency from incident laser energy to thermal energy, and *A_750,915_* is the absorbance of the 750 nm and 915 nm tuned Toco-Photoxil.

Furthermore, *Q_Surr_* is linear with temperature for the outgoing thermal energy, as given by equation (3):

$Q_{Surr}=hS\left( T-T_{Surr} \right)$ (3)

where *h* is heat transfer coefficient, *S* is the surface area of the container, *T* is the temperature, and *T_Surr_* is ambient temperature of the surroundings.

Once the laser power is defined, the heat input *Q_NS_* + *Q_Dis_* will be finite. Since the heat output *Q_Surr_* is increased along with the increase of the temperature according to the equation (3), the system temperature will rise to a maximum when the heat input is equal to heat output as shown in equation (4):

$Q_{NS}+Q_{Dis}=Q_{Surr-Max}=hS\left( T_{Max}-T_{Surr} \right)$ (4)

Where *Q_Surr-Max_* is heat conduction away from the system when the sample cell reaches the equilibrium temperature, and *T_Max_* is the equilibrium temperature. Photothermal transduction efficiency *ƞ* can be calculated by substituting equation (2) for *Q_NS_* into equation (4) and obtaining equation (5):

$ƞ=\frac{hS\left( T_{Max}-T_{Surr} \right)-Q_{Dis}}{I(1-{10}^{-A_{750,915}})}$ (5)

Where *Q_Dis_* was measured independently to be 39.55 mW for 750 nm and 915 nm laser using water, and *(T_Max_ – T_Surr_)* was determined to be 21.8 °C for 750 nm tuned Toco-Photoxil and 15 °C for 915 nm tuned Toco-Photoxil, *I* is 650 mW/cm^2^, A_750_ and A_915_ are the absorbance (0.8497 and 0.5321) of Toco-Photoxil at 750 nm and 915 nm respectively. Thus, only the *hS* remains unknown for calculating *η*. To determine *hS*, a dimensionless driving force temperature, *θ*, is introduced using the maximum system temperature, *T_Max_*_._

$\theta=\frac{T-T_{Surr}}{T_{Max}-T_{Surr}}$ (6)

And a sample system time constant τ_s_

$\tau_{s}=\frac{\sum_{i} m_{i}C_{p,i}}{hS}$ (7)

which is substituted into equation (1) and rearranged to yield

$\frac{d\theta}{dt}=\frac{1}{\tau_{s}}\left[ \frac{Q_{NS}+Q_{Dis}}{hS(T_{Max}-T_{Surr})}- \theta\right]$ (8)

When the laser source was turned off at the cooling stage of the aqueous dispersion of the Toco-Photoxil, Q_NS_ + Q_Dis_ = 0, thereby reducing the equation (8) to

$\frac{d\theta}{dt}=\frac{-\theta}{\tau_{s}}$ (9)

and after integration, giving the expression

$t=-\tau_{s}ln\theta$ (10)

After that, the time constant (τ_s_) was determined by plotting the time versus the negative logarithm of temperature in cooling period. Thus, with the help of equation (10) and (7), *hS* deduced to be 8.4 mW for 750 nm tuned Toco-Photoxil and 9 mW for 915 nm tuned Toco-Photoxil. Also, *m* is 0.3 g and *C* is 4.2 J/g. Substituting these values in equation (5), the photothermal transduction efficiency (*ƞ*) for 750 nm and 915 nm tuned Toco-Photoxil was found to be 25.6 and 20.8% respectively.

**Supplementary tables and figures:**

**Table 1: Abbreviations of different conjugates used in the study**

| **Conjugates** | **Abbreviation** |
| --- | --- |
| Vitamin E modified Gold PLGA nanohybrid | Toco-Photoxil |
| Vitamin E modified Gold PLGA nanohybrid tuned at 750 nm | Toco-Photoxil (750) |
| Vitamin E modified Gold PLGA nanohybrid tuned at 915 nm | Toco-Photoxil (915) |
| Folic acid conjguated Vitamin E modified gold PLGA nanohybrid | FA-Toco-Photoxil |
| IR780 tagged Vitamin E modified gold PLGA nanohybrid | IR780-Toco-Phoxil |
| Folic acid conjguated and IR780 tagged Vitamin E modified gold PLGA nanohybrid | FA-IR780-Toco-Photoxil |

**
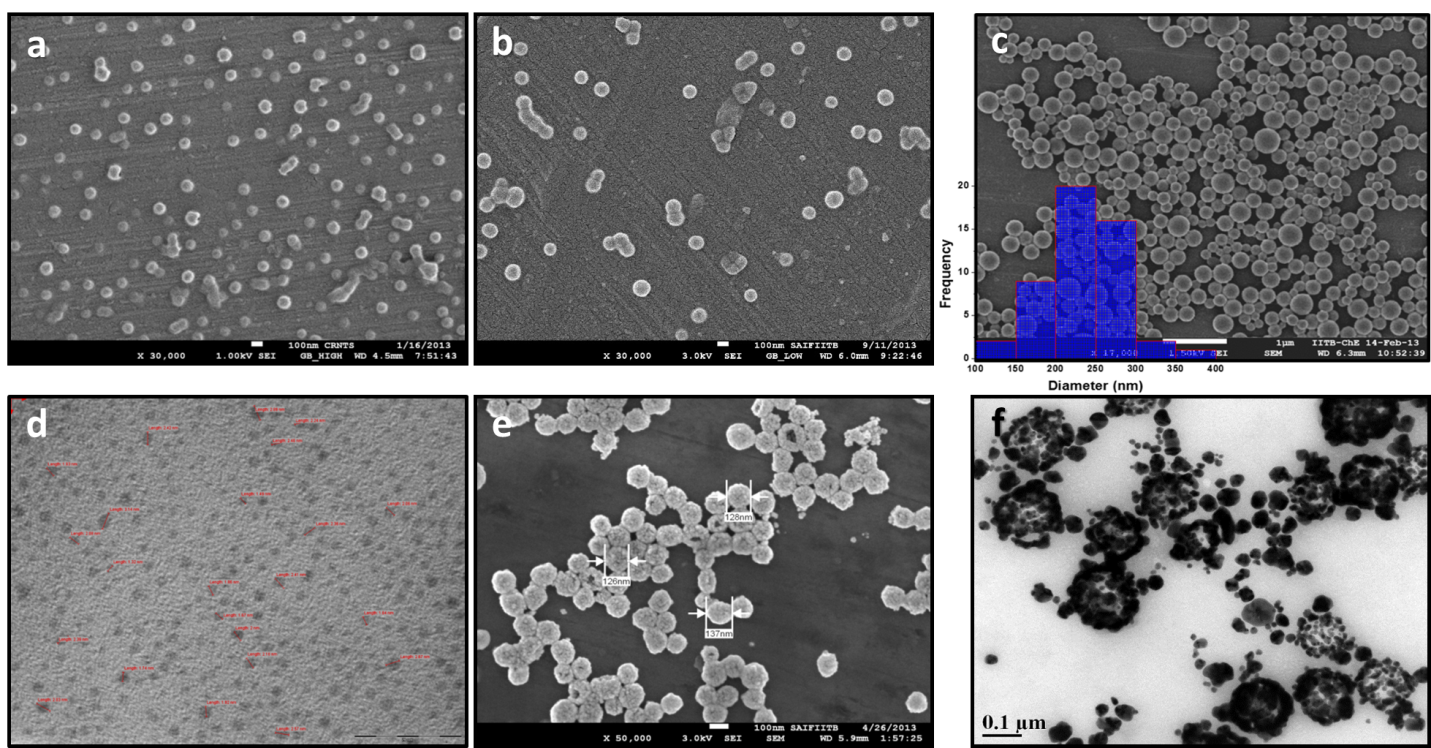
**

**Supplementary Figure S1:** Uncropped FEG-SEM and FEG-TEM images of nanoparticles during the formation and disintegration of Toco-Photoxil. **(a)** FEG-SEM image of PLGA nanoparticles (Scale bar – 100 nm). **(b)** FEG-SEM image of glycol chitosan functionalized PLGA nanoparticles (Scale bar – 100 nm). **(c)** FEG-SEM image of chitosan functionalized PLGA nanoparticles and its size distribution (Scale bar – 1 µm). **(d)** FEG-TEM image of gold seeds formed using THPC (Scale bar – 10 nm). **(e)** FEG-SEM image of Toco-Photoxil (Scale bar – 100 nm). **(f)** FEG-TEM image of disintegrated Toco-Photoxil (Scale bar – 100nm).


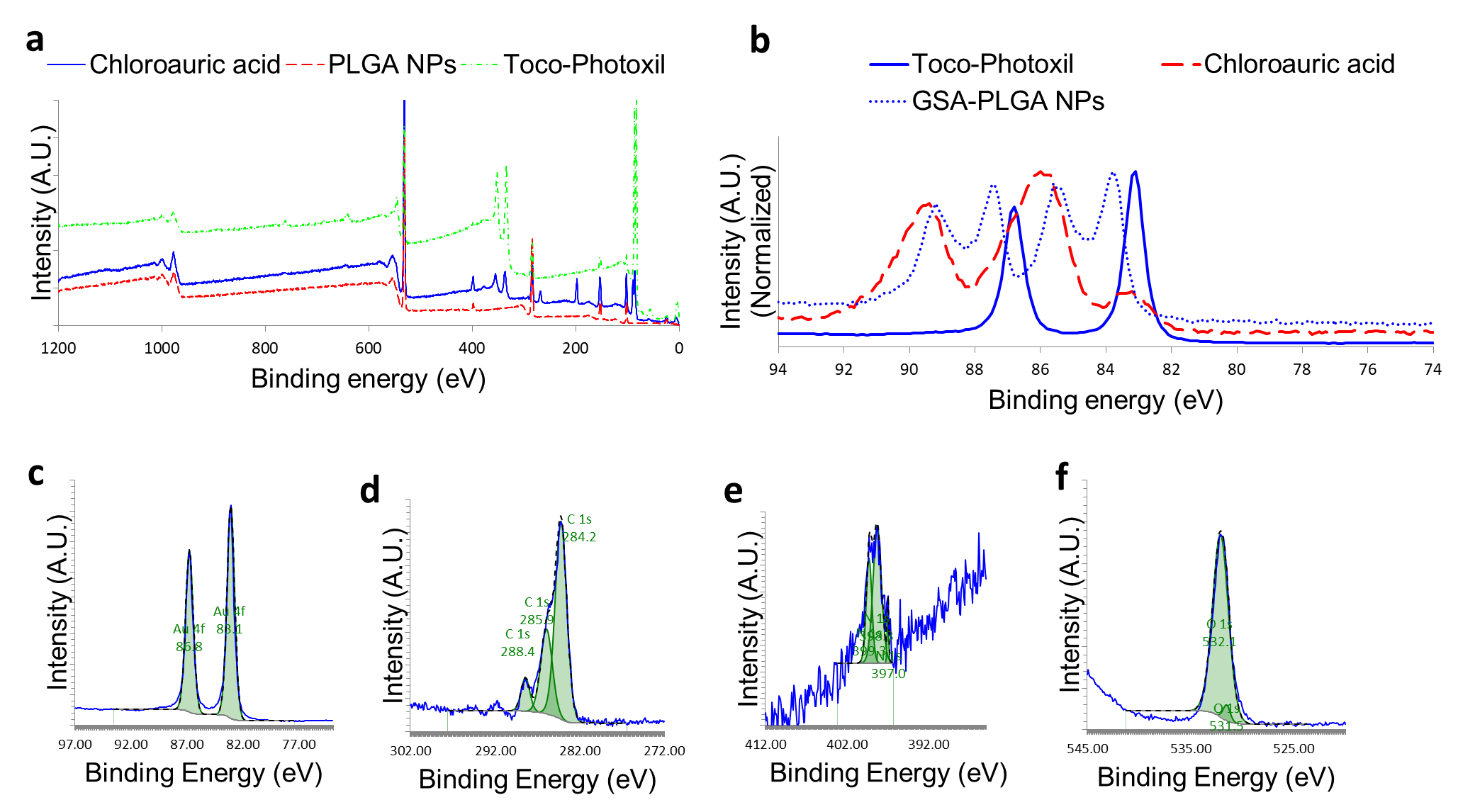


**Supplementary Figure S2: (a)** XPS survey spectra of chloroauric acid, PLGA NPs and Toco-Photoxil. **(b)** Comparative high resolution XPS spectra of Au in chloroauric acid, Toco-Photoxil and gold seeds attached PLGA. **(c.i, ii, iii, iv)** High resolution XPS spectra of Au 4f (i), C 1s (ii), N 1s (iii) and O 1s (iv) of Toco-Photoxil.

**
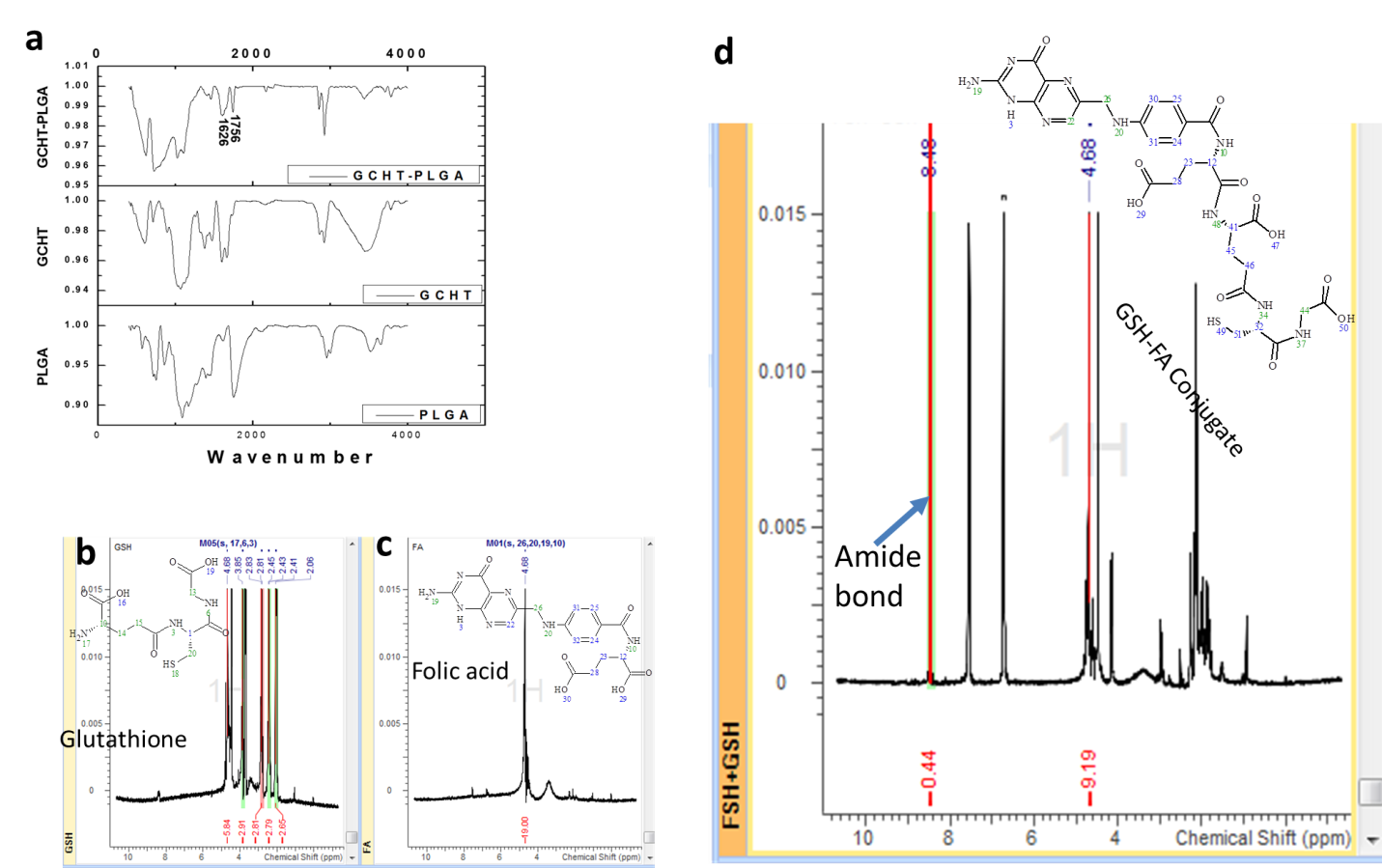
**

**Supplementary Figure S3:** Confirmation of glycol chitosan functionalization over PLGA, and conjugation of glutathione with folic acid using FTIR and NMR respectively. **(a)** FTIR Spectra of GCHT-PLGA, GCHT and PLGA. **(b)** 1H NMR of glutathione. **(c)** Folic acid conjugate. **(d)** Glutathione conjugated folic acid via amide bond.

**
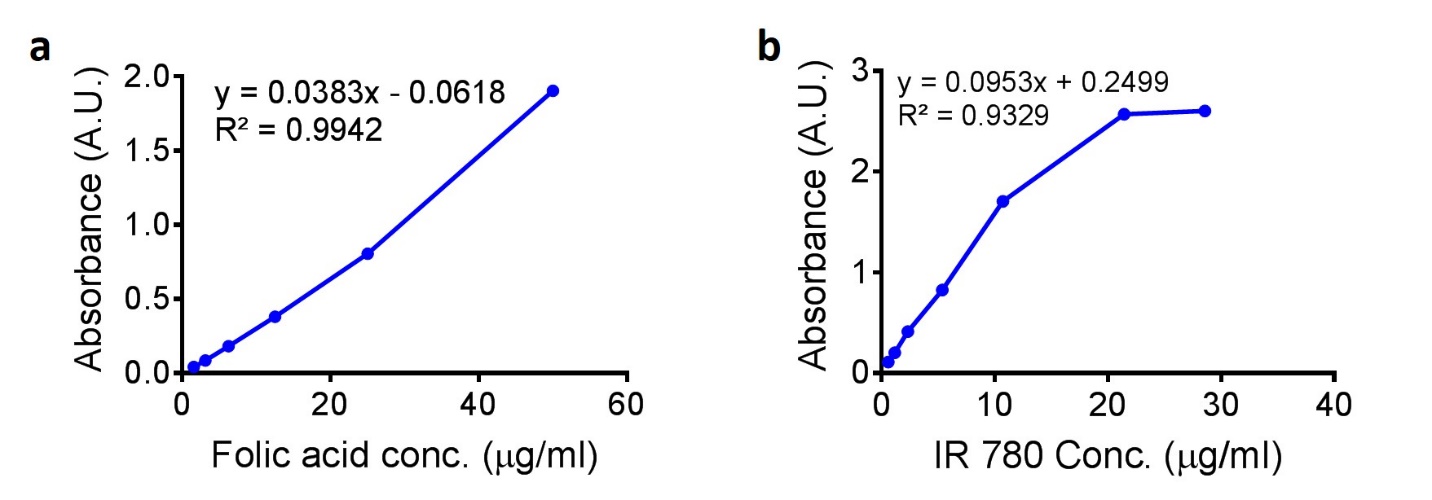
**

**Supplementary Figure S4:** Calibration curve to determine the amount of folic acid and IR780 attached over Toco-Photoxil and FA-Toco-Photoxil. **(a)** Calibration curve of folic acid obtained at 366 nm. **(b)** Calibration of IR780 obtained at 773 nm.

**Calculation to determine number of folic acid molecules attached to Toco-Photoxil:**

**Number of folic acid molecules:**

Number of moles of folic acid = (27x10^-6) / 441.4

= 6.11 X 10^-4

Number of FA molecules =6.11 X 10^-4 X 6.022 X 10^^23^

= 3.6 X 10^16

**Number of Toco-photoxil:**

Mass of gold taken for conjugation: 150 X 10^-6 g

Density of gold= 19.32 g/cm^3^

Volume of gold used= 7.72 X 10^-6 cm^3^

Average size of Toco-Photoxil = 120 nm

Volume of single Toco-Photoxil = 4/3πr^3^

= 4/3 X 3.14 X 60 X10^-7

= 9.04 X 10^-16 cm^3^

Number of Toco-Photoxil = Volume of gold used/Volume of nanoparticle

= 7.77 X 10^-6/ 9.04 X 10^-16

= 8.597 X 10^9

**Number of FA molecules per Toco-Photoxil**

= 3.6 X 10^16 / 8.597 X 10^9

= 4.1 X 10^6

**
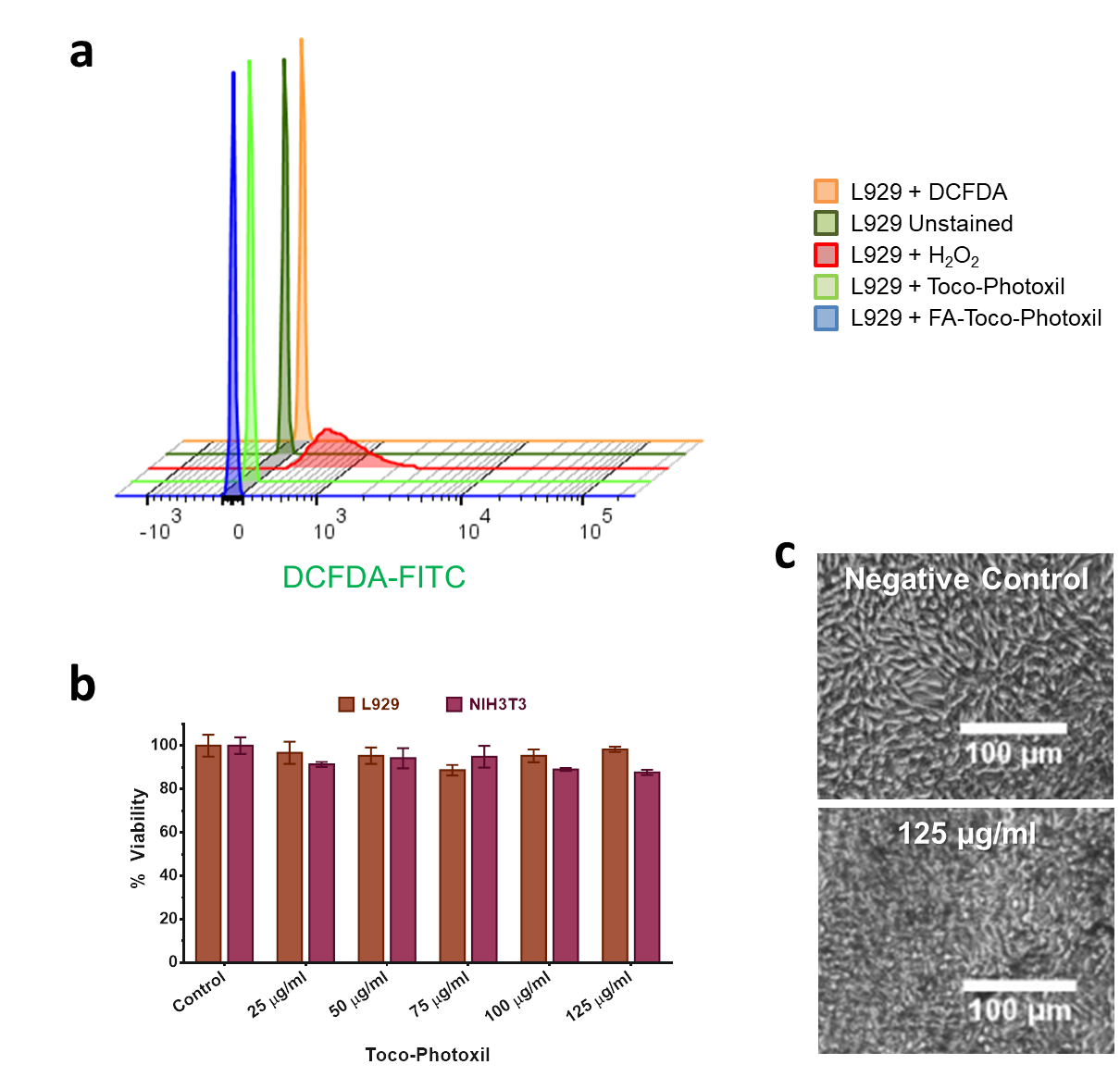
**

**Supplementary Figure S5:** *In vitro* assessment of Toco-Photoxil biocompatibility using DCFDA and MTT assay. **(a)** Histogram profile plot showing H2DCFDA fluorescence of L929 cells treated with H_2_O_2_, Toco-Photoxil_,_ and FA-(GSH)-Toco-Photoxil. **(b)** Percentage cell viability of L929 and NIH3T3 cells determined using MTT assay. **(c)** Corresponding bright field microscopic images of L929 cells.

**
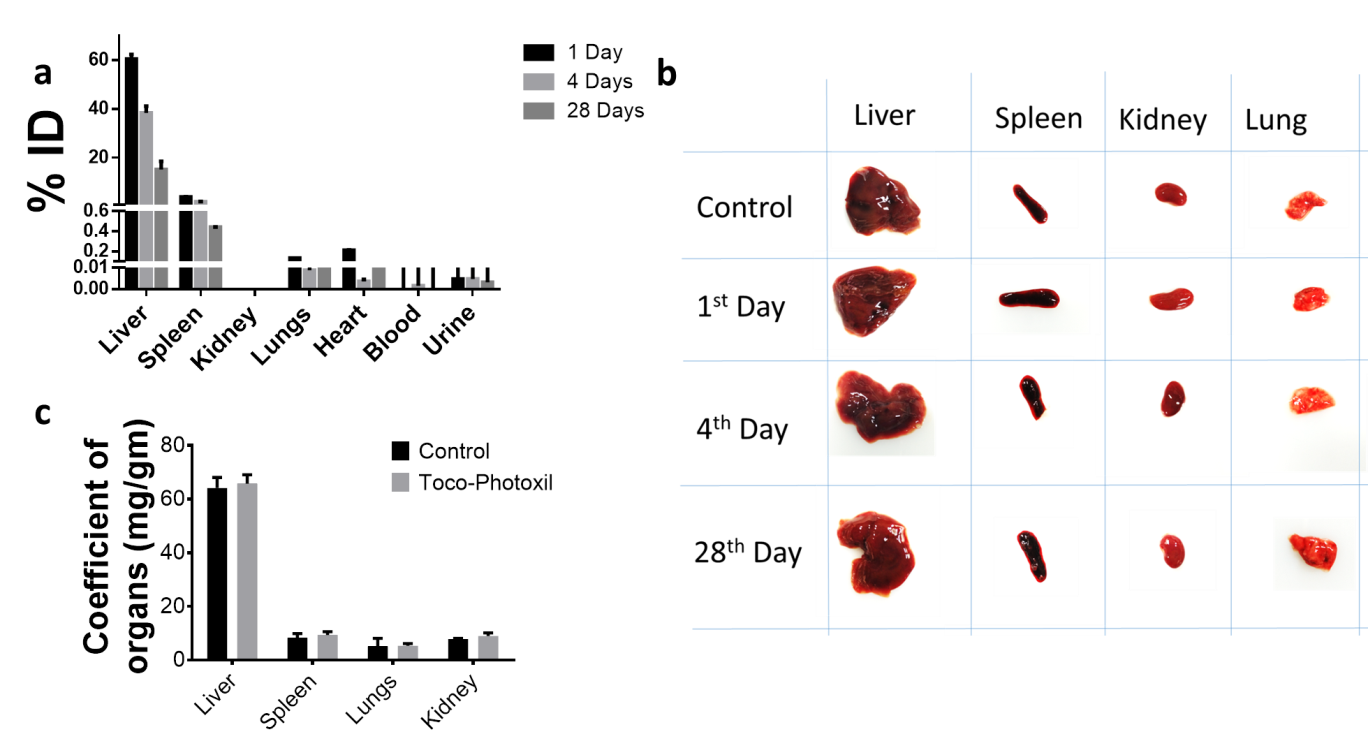
**

**Supplementary Figure S6:** Biodistribution of Toco-Photoxil and gross pathological examination of major organs. **(a)** Gold concentration determined using ICP-MS in terms of % of injection dosage in major organs harvested at 1, 4 and 28 days after intravenous injection. **(b)** Gross pathological examination of major organs harvested at 1, 4 and 28^th^ day of biodistribution study. **(c)** Coefficient of organs determined after 28 days of Toco-Photoxil administration, as per ratio of organs (mg) to mouse body weight (gms).

**
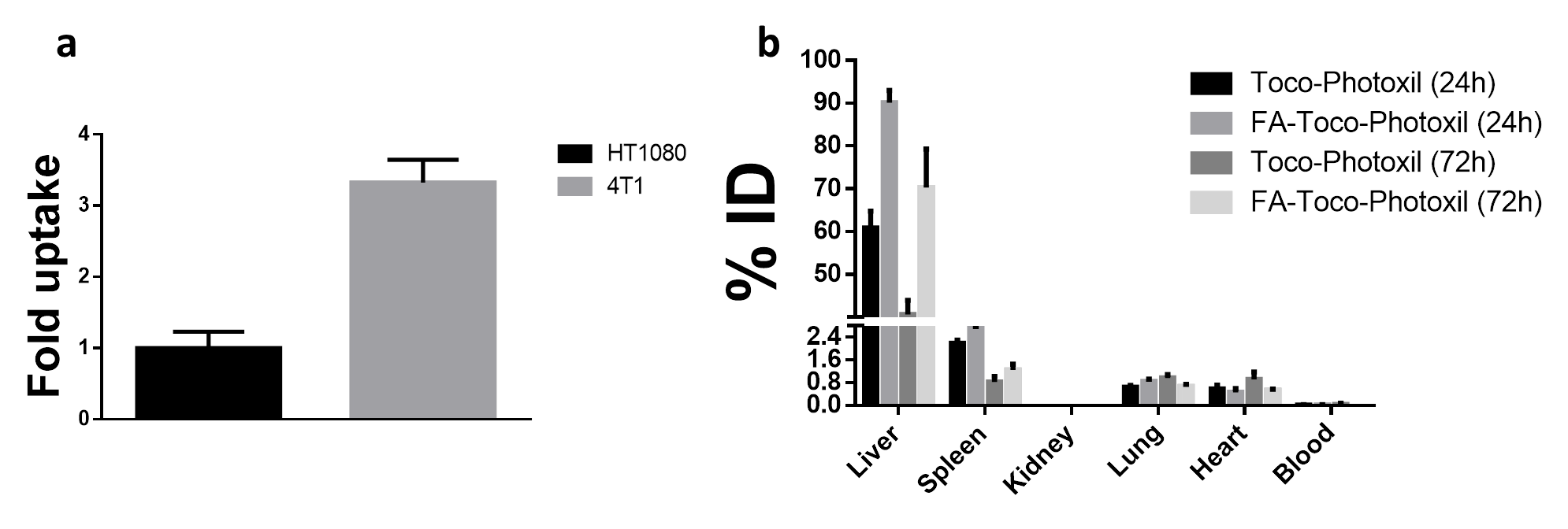
**

**Supplementary Figure S7:** Comparative uptake of targeted and non-targeted Toco-Photoxil. **(a)** Fold uptake of Toco-Photoxil in folate receptor positive (4T1) and folate receptor negative (HT1080) cell lines. **(b)** The percentage accumulation of Toco-Photoxil and FA-Toco-Photoxil in major organs at different time period.

**
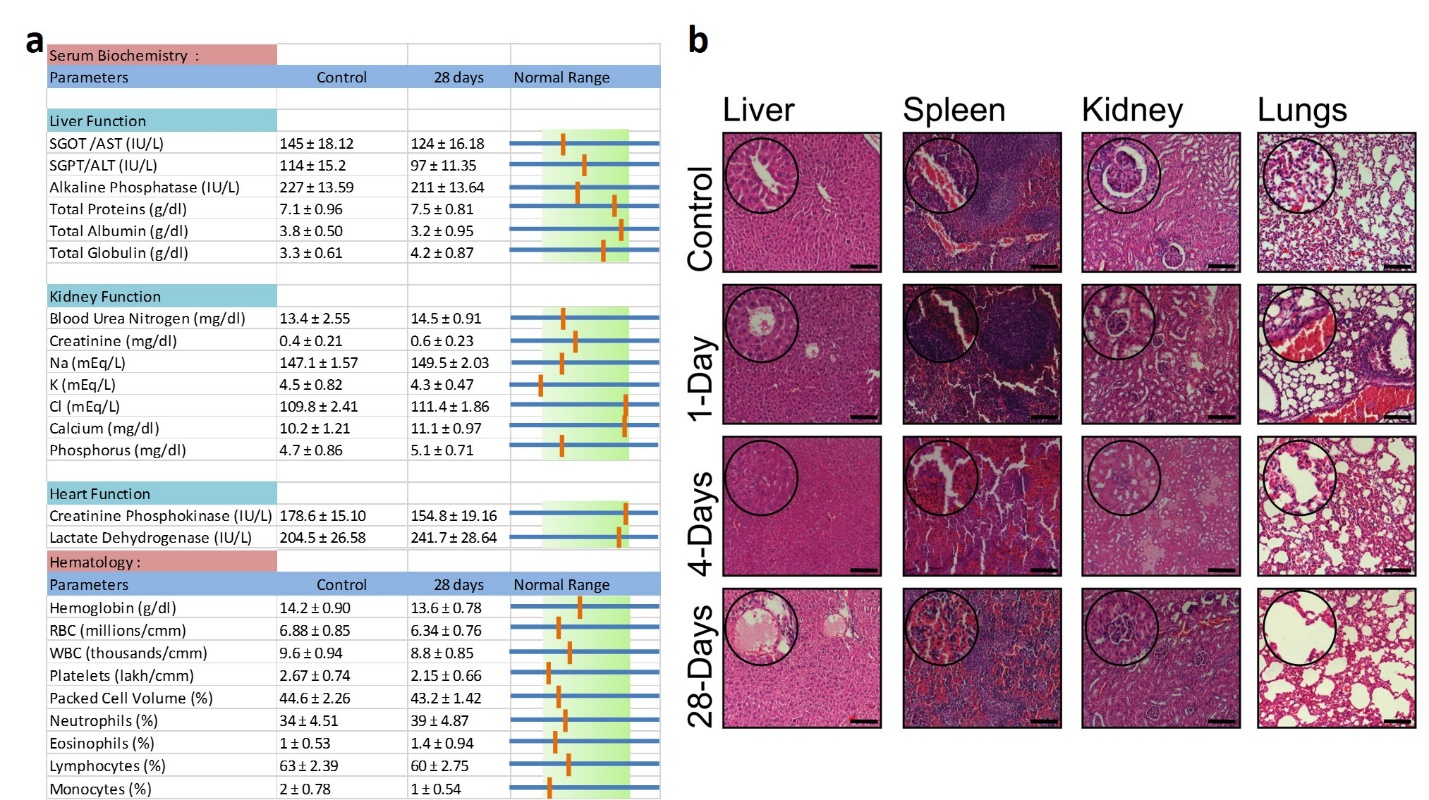
**

**Supplementary Figure S8:** *In vivo* assessment of Toco-Photoxil biocompatibility. **(a)** Serum biochemical analysis and hematology of blood taken from the mice sacrificed on 28^th^ day (end point determination). **(b)** Histopathology of major organs harvested at 28^th^ day of study (Scale bar - 100 µm; zoom factor, 2x); there was no apparent signs of overt microscopic pathological changes between the control and test samples.


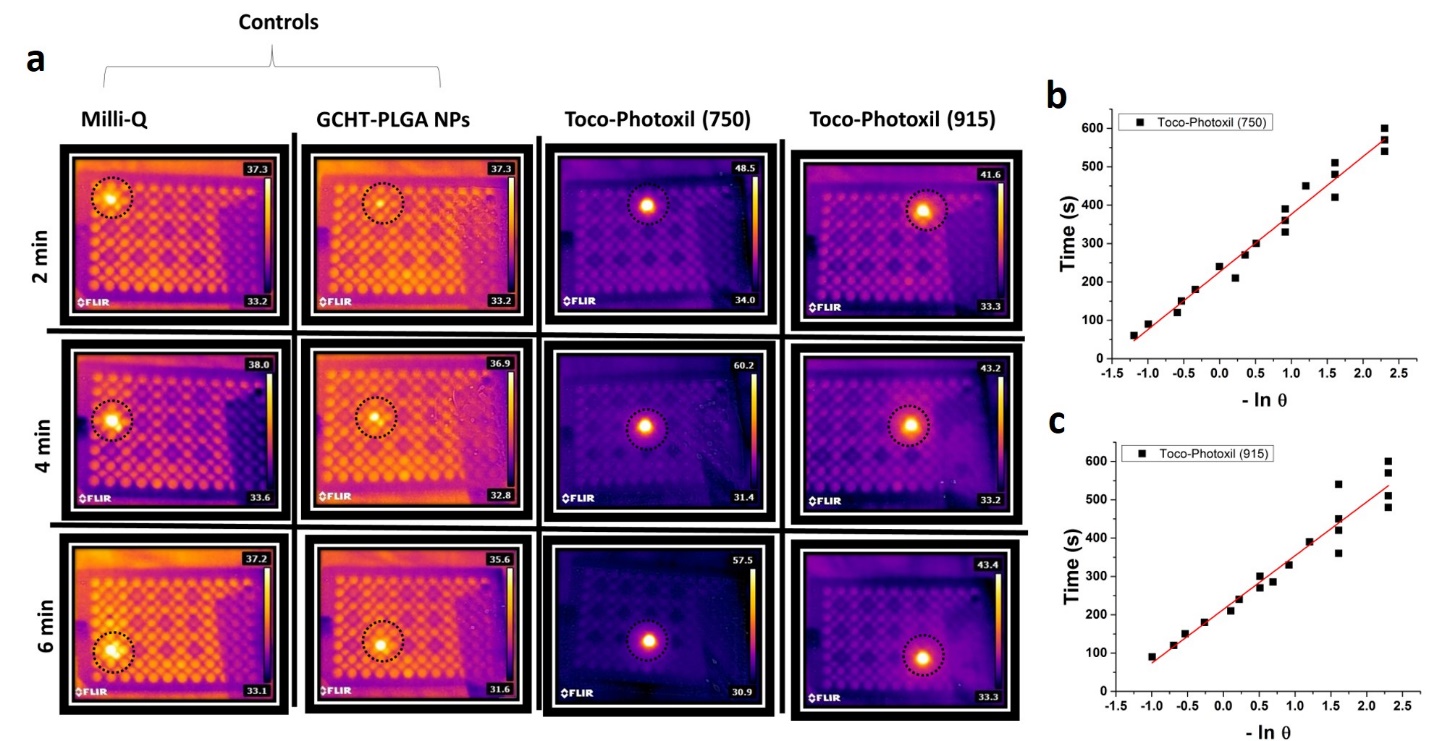


**Supplementary Figure S9:** **(a)** Photographic images of temperature raise by water, PLGA NPs, Toco-Photoxil (750) and Toco-Photoxil (915) determined using thermal imaging camera (FLIR E5). **(b & c)** Time versus negative natural logarithm of driving force temperature from the cooling stage of Toco-Photoxil (750) & (915).


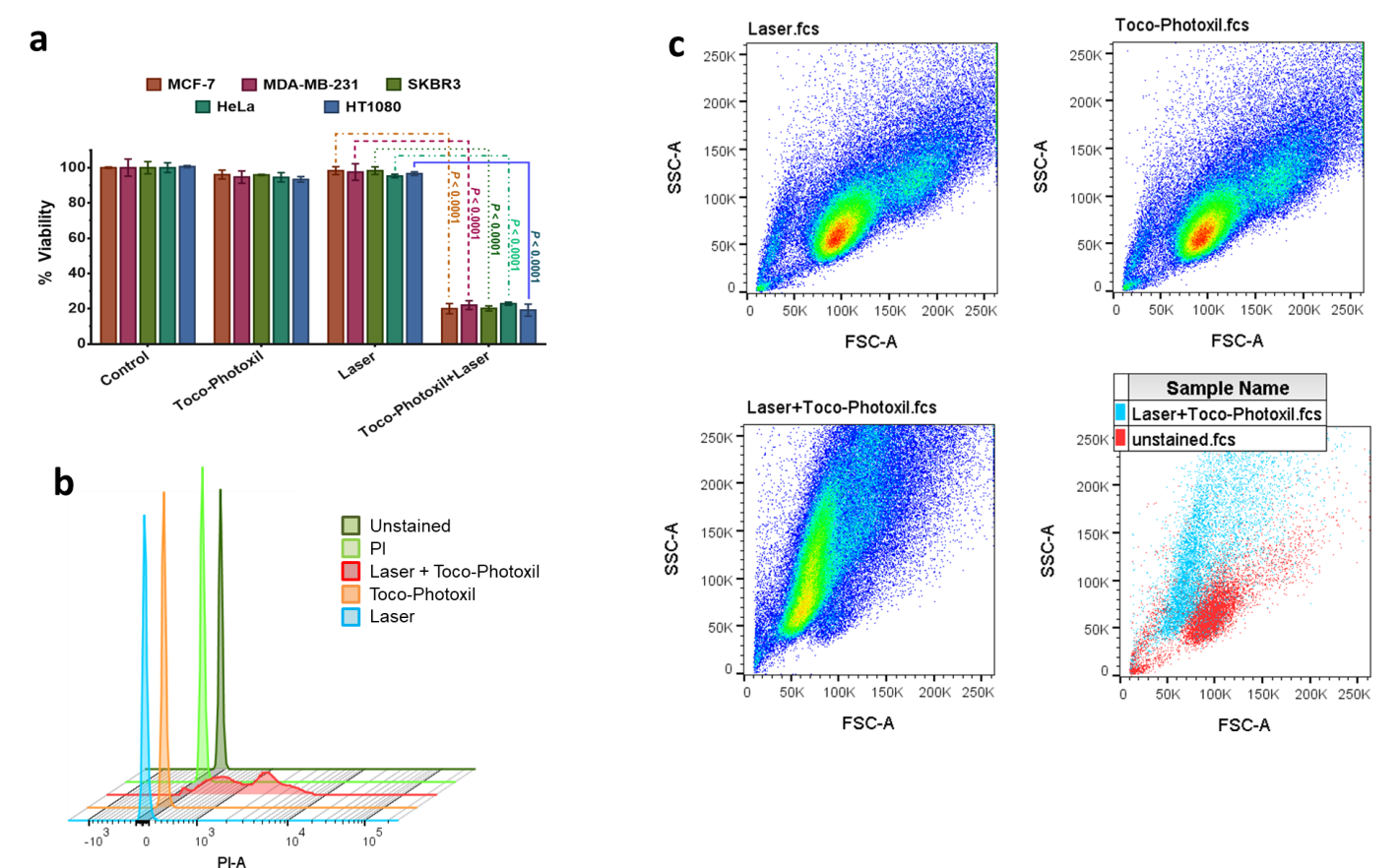


**Supplementary Figure S10:** *In vitro* assessment of Toco-Photoxil directed photothermal therapy in various cancer cell lines using MTT assay and propidium iodide. **(a)** Percentage cell viability determined using MTT assay after *in vitro* PTT over MCF-7, MDA-MB231, SKBR-3, HeLa, and HT1080. **(b)** Histogram profile plot showing PI fluorescence in MCF-7 cells treated with Laser, Toco-Photoxil, and Laser + Toco-Photoxil. **(c)** FSC vs SSC of MCF-7 cells treated with Laser, Toco-Photoxil and Laser + Toco-Photoxil


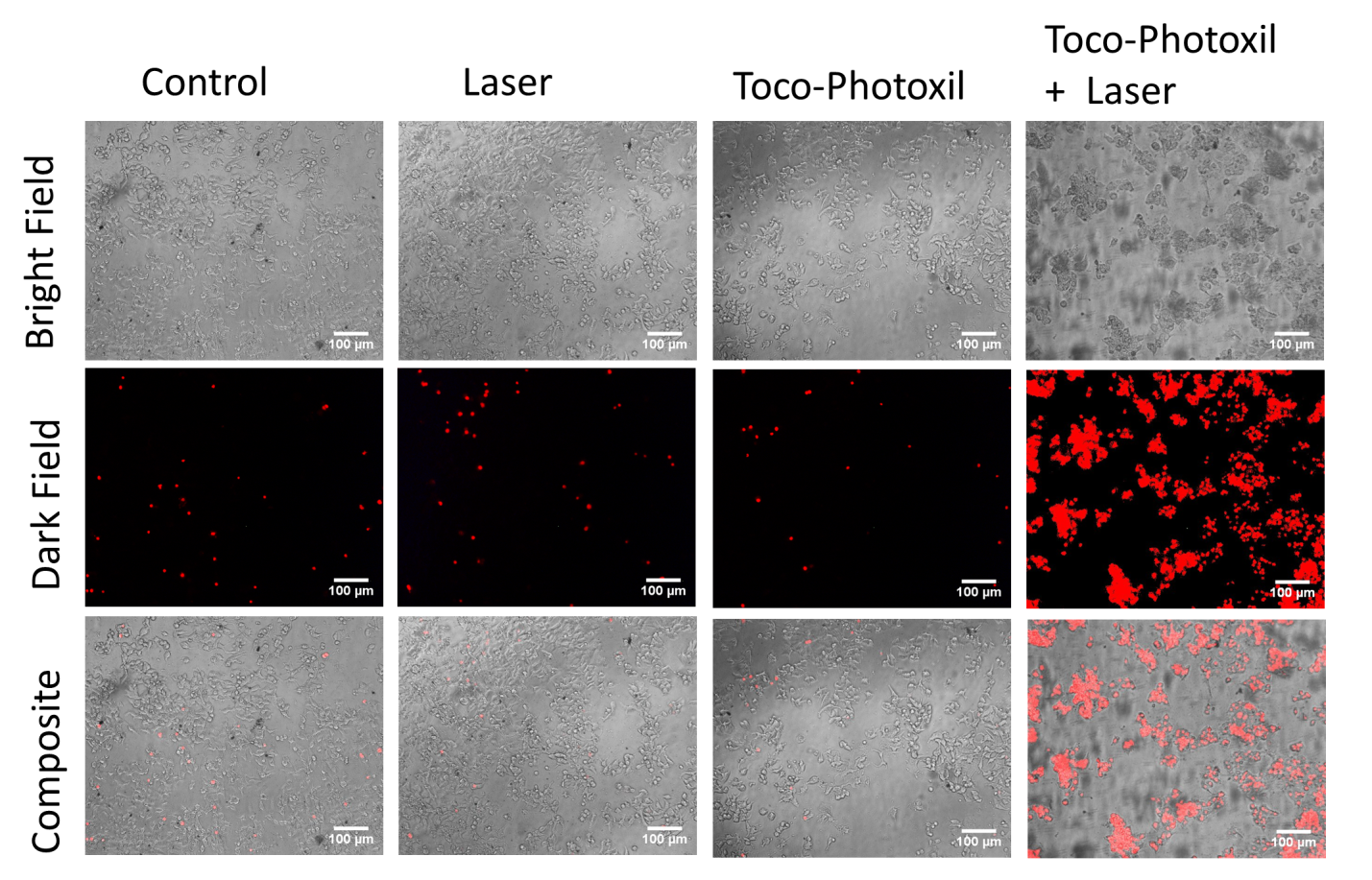


**Supplementary Figure S11:** MCF-7 microscopic images after PTT; red stain due to uptake of PI by dead cells


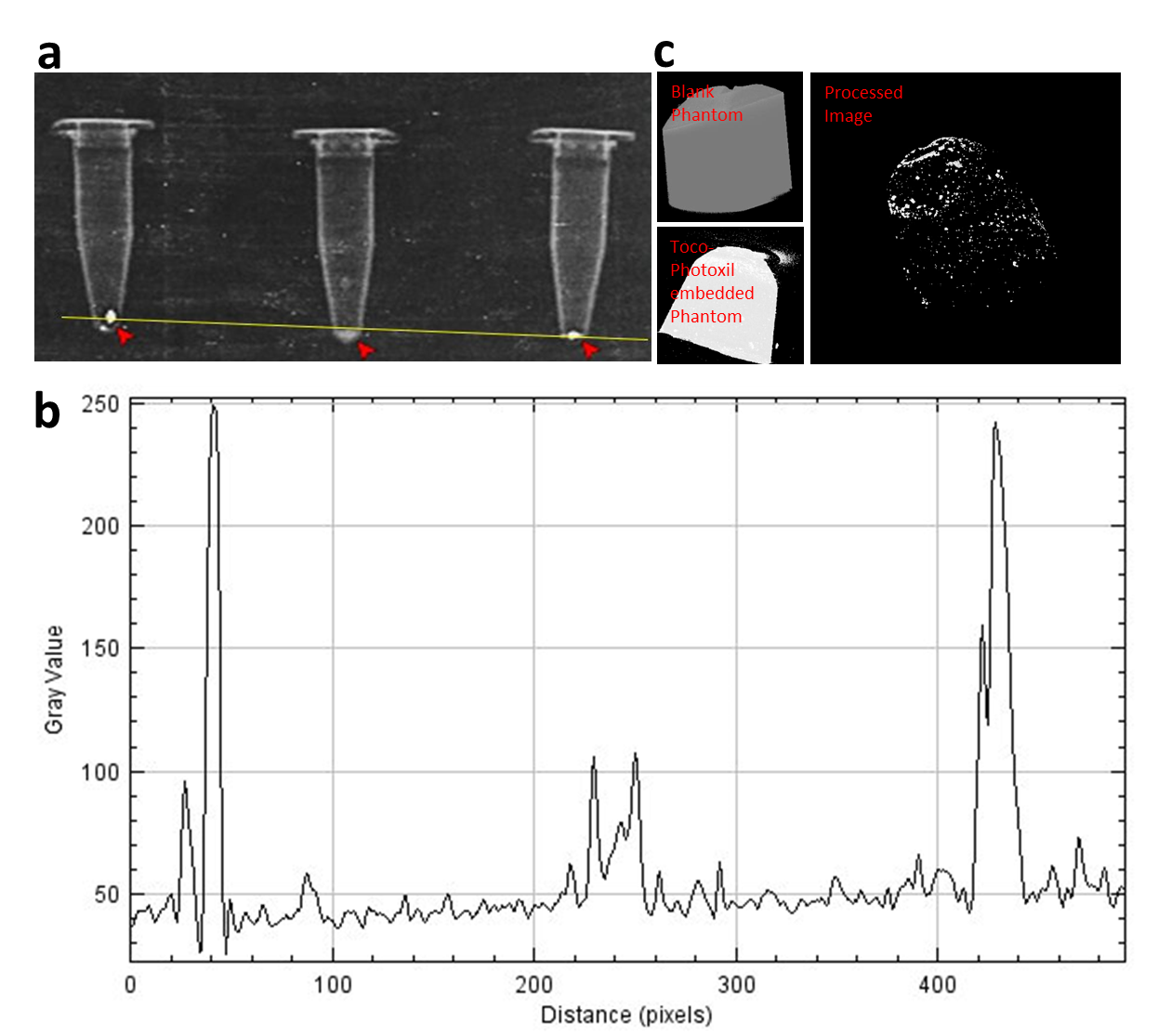


**Supplementary Figure S12:** Diagnostic radiology potential of Toco-Photoxil assessed with the help of X-ray imaging and FDXM. **(a)** X-ray imaging of tubes containing Toco-Photoxil, water and iodine (left to right) where iodine was used as a positive contrast agent and water as negative contrast agent. **(b)** Plot profile to determine the exact gray value in the X-ray image. **(c)** FDXM images of blank phantom, Toco-Photoxil embedded phantom, and a processed image using ImageJ and Fiji.

**REFERENCES**

1. Rao, J. P. & Geckeler, K. E. Polymer nanoparticles: Preparation techniques and size-control parameters. *Prog. Polym. Sci.* **36,** 887–913 (2011).

2. Pham, T., Jackson, J. B., Halas, N. J. & Lee, T. R. Preparation and characterization of gold nanoshells coated with self-assembled monolayers. *Langmuir* **18,** 4915–4920 (2002).

3. Graf, C. & van Blaaderen, A. Metallodielectric colloidal core−shell particles for photonic applications. *Langmuir* **18,** 524–534 (2002).

4. Duff, D. G., Baiker, A., Gameson, I. & Edwards, P. P. A new hydrosol of gold clusters. 2. A comparison of some different measurement techniques. *Langmuir* **9,** 2310–2317 (1993).

5. Roper, D. K., Ahn, W. & Hoepfner, M. Microscale heat transfer transduced by surface plasmon resonant gold nanoparticles. *J. Phys. Chem. C. Nanomater. Interfaces* **111,** 3636–3641 (2007).
